# Dynein/Dynactin-Mediated Transport of Kinetochore Components off Kinetochores and onto Spindle Poles Induced by Nordihydroguaiaretic Acid

**DOI:** 10.1371/journal.pone.0016494

**Published:** 2011-01-28

**Authors:** Jakub K. Famulski, Larissa J. Vos, Jerome B. Rattner, Gordon K. Chan

**Affiliations:** 1 Department of Oncology, University of Alberta, Edmonton, Alberta, Canada; 2 Department of Anatomy and Cell Biology, University of Calgary, Calgary, Alberta, Canada; 3 Experimental Oncology, Cross Cancer Institute, Edmonton, Alberta, Canada; 4 School of Cancer, Engineering and Imaging Sciences, University of Alberta, Edmonton, Alberta, Canada; University of Edinburgh, United Kingdom

## Abstract

The mitotic checkpoint functions to ensure accurate chromosome segregation by regulating the progression from metaphase to anaphase. Once the checkpoint has been satisfied, it is inactivated in order to allow the cell to proceed into anaphase and complete the cell cycle. The minus end-directed microtubule motor dynein/dynactin has been implicated in the silencing of the mitotic checkpoint by “stripping” checkpoint proteins off kinetochores. A recent study suggested that Nordihydroguaiaretic acid (NDGA) stimulates dynein/dynactin-mediated transport of its cargo including ZW10 (Zeste White 10). We analyzed the effects of NDGA on dynein/dynactin dependent transport of the RZZ (Zeste White 10, Roughdeal, Zwilch) complex as well as other kinetochore components from kinetochores to spindle poles. Through this approach we have catalogued several kinetochore and centromere components as dynein/dynactin cargo. These include hZW10, hZwilch, hROD, hSpindly, hMad1, hMad2, hCENP-E, hCdc27, cyclin-B and hMps1. Furthermore, we found that treatment with NDGA induced a robust accumulation and complete stabilization of hZW10 at spindle poles. This finding suggests that NDGA may not induce dynein/dynactin transport but rather interfere with cargo release. Lastly, we determined that NDGA induced accumulation of checkpoint proteins at the poles requires dynein/dynactin-mediated transport, hZW10 kinetochore localization and kinetochore-microtubule attachments but not tension or Aurora B kinase activity.

## Introduction

Accurate segregation of chromosomes during mitosis is required for the maintenance of genomic stability. Failure or improper execution of mitosis is catastrophic for individual cells as well as a potential precursor to malignancy. The mis-segregation of even one chromosome can negatively impact cell survival or conversely lead to mis-regulation of cell growth. Numerous human cancers have been associated with elevated levels of aneuploidy that are thought to result from chromosome mis-segregation (for a review see reference[1]). In order to avoid aneuploidy, a surveillance mechanism, the mitotic checkpoint, monitors and ensures accurate chromosome segregation. The mitotic checkpoint ensures accurate chromosome segregation by preventing the progression from metaphase into anaphase (reviewed in[2], [3]). In general, the checkpoint arrests cells in mitosis until all chromosomes have aligned at the metaphase plate. Chromosome alignment depends on the attachment of microtubules (MTs) emanating from spindle poles to kinetochores on chromosomes (reviewed in[4]). As such, the checkpoint directly monitors for kinetochore-MT (k-MT) attachments and initiates mitotic arrest in their absence. The mitotic checkpoint directly inhibits the Anaphase Promoting Complex/Cyclosome (APC/C), an E3 ubiquitin ligase, which is responsible for targeting cyclin B and securin for degradation through the 26S proteasome.[5], [6] Inhibition of the APC/C ensures that sister chromatids remain physically connected and that Cdk1 activity remains high. All known essential components of the mitotic checkpoint localize to kinetochores in response to mitotic checkpoint signaling.[3] However, certain kinetochore checkpoint proteins are also known to transiently localize to spindle poles through dynein/dynactin-mediated transport off kinetochores and along k-MTs.[7] Moreover, the APC/C as well as cyclin B are known to reside on spindle poles during mitosis and cyclin B degradation during the metaphase-anaphase transition occurs specifically at spindle poles and the mitotic spindle.[8], [9], [10] The localization of mitotic checkpoint components on the spindle and spindle poles is therefore an essential component of mitotic checkpoint signaling and silencing.

It has been recently shown that treatment with the small molecule Nordihydroguaiaretic acid (NDGA) results in the accumulation of human *Zeste White 10* (hZW10) at centrosomes and spindle poles.[11] hZW10 is a component of the evolutionarily conserved Roughdeal (hROD), ZW10, Zwilch (RZZ) complex that is known to transport along k-MTs off kinetochores and onto spindle poles via dynein/dynactin.[12], [13], [14], [15] Furthermore, the RZZ complex is an essential component of the mitotic checkpoint whose kinetochore residency dynamics regulate its function.[16], [17] hZW10 and hROD are known to transiently localize to spindle poles during prometaphase and metaphase,[13], [18], [19] however, the amount of hZW10 associated with the spindle poles appears significantly increased in the presence of NDGA.[11] Initial studies of NDGA showed that it can enhance the interaction between dynein/dynactin and its cargo, such as hZW10, although the molecular mechanism of its action remains unknown.[11] In our current study we used NDGA to examine the transport of the RZZ complex off kinetochores and onto spindle poles. Furthermore, we also characterized several kinetochore and centromere components for their ability to be transported by dynein/dynactin in the presence of NDGA. Our results indicate that transport of the RZZ complex requires k-MT attachments (monopolar or bi-polar), but not tension or Aurora B kinase function. Furthermore, we find that NDGA treatment results in the stabilization of EGFP-hZW10 at spindle poles suggesting that NDGA may interfere with the release of dynein/dynactin cargo at spindle poles. Lastly, we find that several well established mitotic checkpoint signaling components are also transported off kinetochores in the presence of NDGA.

## Results

### hZW10 is a dynamic resident of mitotic spindle poles

**Figure 1:**
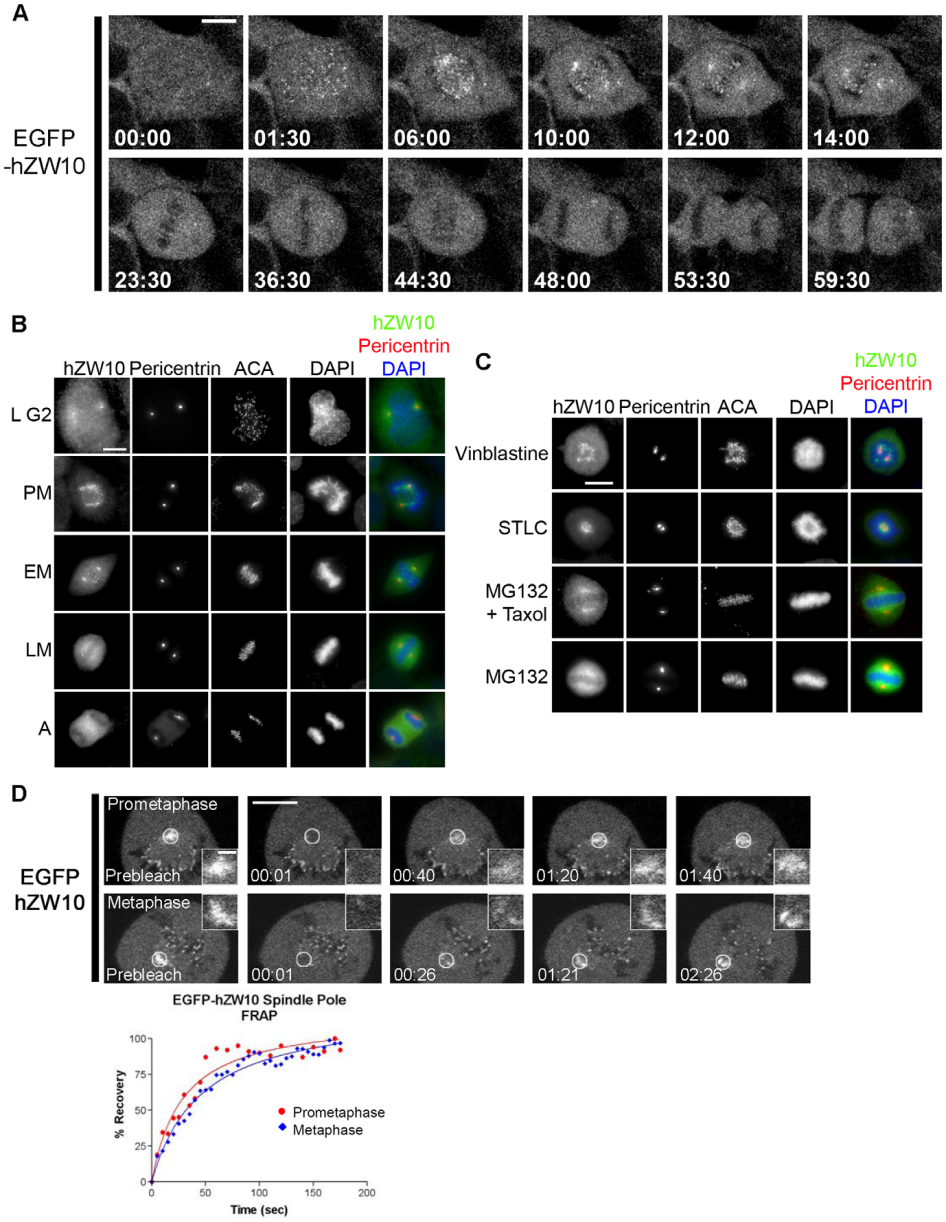
hZW10 transiently localizes to spindle poles during mitosis. **A**) HeLa cells stably expressing EGFP-hZW10 were imaged using a spinning disk confocal microscope. Selected maximum projections of 20 1 µm Z-stacks collected every 30 seconds are displayed. EGFP-hZW10 is observed to first localize to kinetochore upon nuclear envelope breakdown in prophase and subsequently to the spindle poles starting in prometaphase. Kinetochore and spindle pole associated EGFP-hZW10 remains until all the chromosomes become aligned in metaphase. Upon anaphase onset, EGFP-hZW10 is not detected at kinetochores or spindle poles. Time is shown in minutes:seconds, scale bar = 10 µm. **B**) HeLa cells in late G2 (L G2) or the different stages of mitosis are stained for hZW10, pericentrin (as a spindle pole marker), and ACA. hZW10 is observed to localize to kinetochores and spindle poles in prometaphase (PM) and early metaphase (EM). hZW10 no longer localizes to the spindle poles during late metaphase (LM) and in anaphase (A). Pericentrin is observed to stain the spindle poles in all stages of mitosis. Chromosomes are stained with DAPI. Scale bar = 10 µm. **C**) HeLa cells treated with 0.5 µM vinblastine, 7 µM STLC, 12.5 µM MG132 and or 12.5 µM MG132 + 1 µM taxol were stained for hZW10 and pericentrin localization. hZW10 is observed to localize to spindle poles in cells treated with STLC and MG132 + taxol. hZW10 was, however, absent from spindle poles of cells treated with vinblastine or MG132 alone. Pericentrin was observed to stain the spindle poles in all of the treated cells. Chromosomes are stained with DAPI. Scale bar = 10 µm. **D**) HeLa cells stably expressing EGFP-hZW10 were analyzed for turnover of EGFP-hZW10 at the spindle poles using FRAP. White circles indicate the spindle pole bleached which is enlarged in the insets. Time-lapse images of the recovery after photobleaching indicate that in both prometaphase (top) and metaphase (bottom) EGFP-hZW10 is dynamic at the spindle poles. On the right is a % recovery graph of EGFP-hZW10 at spindle poles showing no difference between the recovery of EGFP-hZW10 at prometaphase (red) or metaphase (blue) spindle poles. Large scale bar = 10 µm, small scale bar = 2 µm.

ZW10 is known to localize to spindle poles during mitosis in human and *Drosophila* cells.[7], [20], [21] In order to analyze the localization pattern of hZW10 at spindle poles in live cells we took advantage of a HeLa cell line stably expressing EGFP-hZW10.[17] Using live cell time-lapse microscopy we observed that EGFP-hZW10 began to accumulate at kinetochores during prophase, immediately after nuclear envelope breakdown, but did not localize to the spindle poles until prometaphase (Figure 1A). Once all the chromosomes were aligned at the metaphase plate, EGFP-hZW10 vacated both the kinetochores and spindle poles. The behavior of EGFP-hZW10 in live cells was consistent with endogenous hZW10 as monitored through immunofluorescence (Figure 1B).

In order to determine the conditions under which hZW10 is able to localize to the spindle poles, HeLa cells were treated with several common inhibitors of mitotic spindle function. These included: vinblastine,[22] to depolymerize microtubules and thus remove k-MT attachment; S-trityl-L-cystine (STLC),[23] an Eg5 kinesin inhibitor to generate monopolar spindles and therefore monopolar k-MT attachments; MG132,[24] to inhibit the proteasome and arrest the cells in late metaphase; as well as MG132 followed by low dose taxol, which inhibits MT dynamics and therefore abolishes tension between fully aligned sister kinetochores.[16] hZW10 only localized to the spindle poles, as confirmed by co-staining with pericentrin, during the STLC and MG132 + taxol treatments (Figure 1C) indicating that spindle pole localization is restricted to checkpoint active cells with established k-MT attachments. Our results indicate that hZW10 spindle pole localization requires k-MT attachments, but the localization is diminished once chromosome alignment and inter-kinetochore tension is achieved. The requirements for hZW10 spindle pole localization findings are similar to the requirements for dynein/dynactin dependent transport of checkpoint proteins off kinetochores.[15], [25]

Lastly, we also analyzed the dynamics of EGFP-hZW10 at spindle poles using the Fluorescence Recovery After Photobleaching (FRAP) technique. We found that EGFP-hZW10 is a dynamic component of the spindle pole during both prometaphase and metaphase with 50% fluorescence recovery times (t^1/2^) of 27.7 +/− 11.1 seconds (n = 30) and 23.9 +/− 8.7 seconds (n = 10) respectively (Figure 1D). The observed behaviour of hZW10 at the spindle pole indicates it is a dynamic component which vacates the spindle pole soon after it arrives there.

### NDGA induces transport, stabilization and accumulation of hZW10 at spindle poles

**Figure 2:**
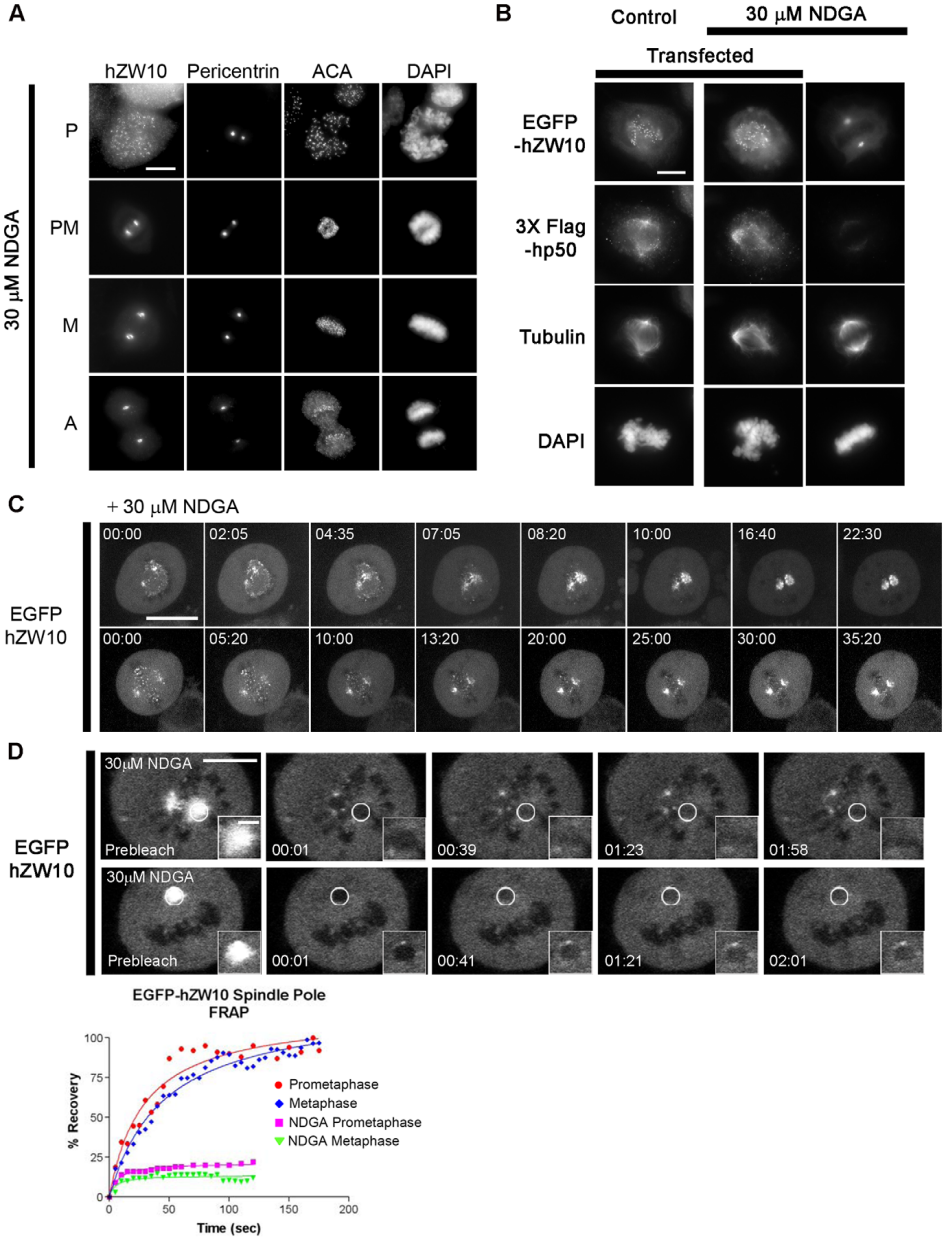
NDGA induced hZW10 accumulation at spindle poles is stable and requires dynein/dynactin dependent transport. **A**) HeLa cells treated with 30 µM NDGA for 30 minutes and stained with hZW10, pericentrin and ACA antibodies. hZW10 localizes to the kinetochore in prophase (P) and co-localizes with pericentrin during prometaphase (PM), metaphase (M) and anaphase (A). Chromosomes are stained with DAPI. Scale bar = 10 µm. **B**) HeLa cells transiently transfected with 3X Flag-hp50 for 24 hours and then treated with 30 µM NDGA for 30 minutes. Coverslips were labeled with antibodies against hZW10, Flag and Tubulin and chromosomes are stained with DAPI. hZW10 was not able to accumulate at spindle poles when dynein/dynactin function is disrupted. Scale bar = 10 µm. **C**) HeLa cells stably expressing EGFP-hZW10 were treated with 30 µM NDGA and immediately imaged using the spinning disk confocal microscope. Maximum projections of ∼20 1 µm Z-stacks are shown. EGFP-hZW10 is transported to the spindle pole within minutes of adding NDGA in both prometaphase (top) and metaphase (bottom). Time shown as minutes:seconds, scale bar = 10 µm **D**) HeLa cells stably expressing EGFP-hZW10 were treated with 30 µM NDGA for 30 minutes and analyzed for turnover of EGFP-hZW10 at the spindle poles using FRAP. White circles indicate the spindle pole bleached which is enlarged in the insets. Time-lapse images of the recovery after photobleaching indicate that in the presence of NDGA EGFP-hZW10 is not dynamic at either prometaphase (top) or metaphase (bottom) spindle poles. The percent recovery graph is shown to the right. Large scale bar = 10 µm, small scale bar = 2 µm.

Recently, it has been shown that NDGA can induce the transport of hZW10 to spindle poles and centrosomes by stabilizing the interaction between hZW10 and dynein/dynactin.[11] Although the effect of NDGA on dynein/dynactin transport has not been investigated directly, the observed effect of NDGA on the accumulation of dynein/dynactin and hZW10 at spindle poles during mitosis is clear.[11] As such, we were interested in investigating the mechanism as well as consequence(s) of hZW10 spindle pole accumulation. To do so, we first confirmed the NDGA phenotype by treating HeLa cells with 30 µM NDGA and analyzing hZW10 and pericentrin localization by immunofluorescence staining. After 30 minutes of NDGA treatment, hZW10 was accumulated at spindle poles in all but prophase mitotic cells (Figure 2A). Moreover, hZW10 was not observed at kinetochores and appeared to be reduced in the cytoplasm in the presence of NDGA. Fluorescence intensity measurements indicated that in the presence of NDGA, hZW10 spindle pole occupancy increased from ∼8.5% of total hZW10 normally found at prometaphase and metaphase spindle poles to ∼20%. This constitutes an approximate 2.35 fold increase of hZW10 at spindle poles and was found to be NDGA concentration dependent (hZW10 pole occupancy of 8.1±2.1% for 10 µM, 11.3±4. 5% for 15 µM and 22.4±2.7% for 30 µM). The spindle pole accumulation of hZW10 is unlikely the result of the lipoxygenase activity of NDGA as two additional lipoxygenase inhibitors, PD 146176 and Caffeic acid phenethyl ester, were tested and did not induce accumulation of hZW10.

**Figure 3:**
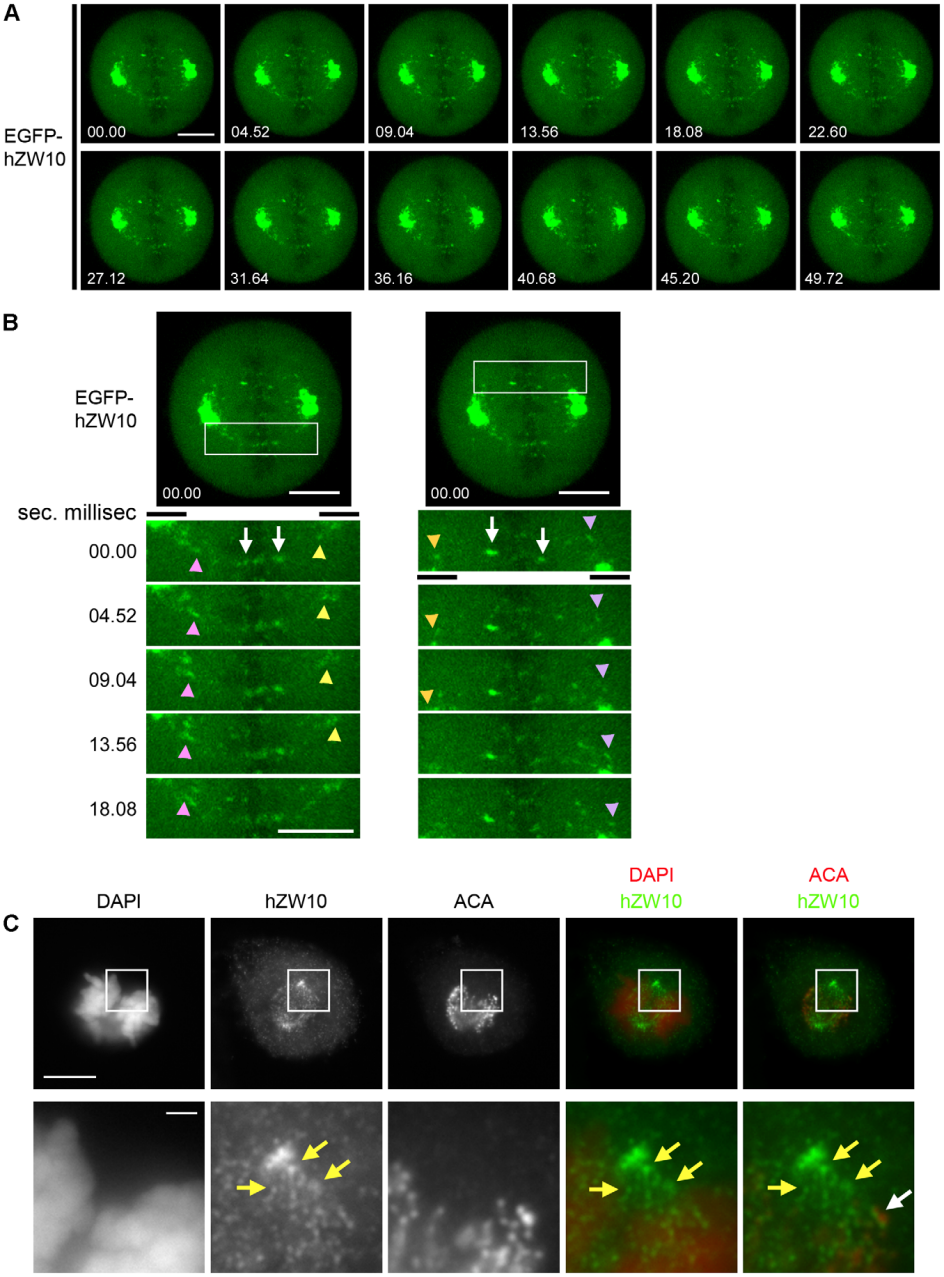
NDGA induced spindle pole accumulation of EGFP-hZW10 occurs by kinetochore ‘shedding’ to the spindle pole. **A**) HeLa cells stably expressing EGFP-hZW10 were treated with 30 µM NDGA imaged 15 minutes after drug addition using a spinning disk confocal microscope. Maximum projections for every second frame of 5 1 µm Z-stacks collected every 2.26 seconds are shown. EGFP-hZW10 had accumulated at the spindle poles at the start of filming and is seen streaming towards the pole from the kinetochore. Time shown as seconds.milliseconds, scale bar = 10 µm **B**) Magnified images from the movie stills showing EGFP-hZW10 foci streaming towards the poles (marked with a black line). The white arrows indicate kinetochore bound EGFP-hZW10 and the coloured arrowheads point to streaming EGFP-hZW10 foci. Time shown as seconds.milliseconds, White scale bars = 10 µm. **C**) HeLa cells treated with 30 µM NDGA for 15 minutes and stained with hZW10 and ACA antibodies. Chromosomes are stained with DAPI. hZW10 foci can be seen between the kinetochores and the spindle poles, indicative of streaming. Magnified views are shown below. The yellow arrows indicate the hZW10 foci that are presumed to be streaming towards the pole and are no longer kinetochore associated (white arrow). Large scale bar = 10 µm, small scale bar = 1 µm.

Having established that NDGA can induce hZW10 accumulation at the spindle poles, we used time-lapse fluorescence microscopy to monitor this in live cells. Using EGFP-hZW10 expressing HeLa cells, we monitored the dynamics of NDGA-induced EGFP-hZW10 transport to the spindle poles in both prometaphase and metaphase (Figure 2C). Our live cell imaging confirmed that NDGA treatment results in the accumulation of EGFP-hZW10 at the spindle poles (Movie S1). The observed accumulation of EGFP-hZW10 began to occur within ∼10 minutes of NDGA addition in both prometaphase and metaphase cells. Examination of cells 15 minutes after NDGA addition by either immunofluorescence or live cell microscopy revealed punctate staining between the kinetochore and the spindle pole indicative of ‘shedding’ along microtubules (Figure 3 and Movie S2). The rate of movement of the EGFP-hZW10 foci towards the spindle pole was calculated to be between 0.098 µm per second and 0.123 µm per second. The rate of transport is higher than previously reported for mammalian cells[26], [27] but lower than that reported for GFP-Rod in syncytial *Drosophila* embryos.[12] We used FRAP to determine whether the NDGA-dependent over-accumulation of hZW10 at spindle poles is a result of changes in the turn-over of the protein at spindle poles. FRAP analysis revealed that, while EGFP-hZW10 was highly dynamic at spindle poles in untreated cells (Figure 1D), NDGA treatment stabilized hZW10 at the spindle pole during both prometaphase and metaphase (Figure 2D). We thus conclude that the accumulation of hZW10 at spindle poles in the presence of NDGA occurs because hZW10 is unable to dissociate from the spindle pole.

Inhibition of dynein/dynactin kinetochore localization by injection of p50/dynamitin has been shown to prevent relocalization of kinetochore proteins Mad2, BubR1 and CENP-E to the spindle pole in ATP depleted cells.[7] To examine if dynein/dynactin was required for the accumulation of hZW10 at the poles, we overexpressed hp50/dynamitin[28] through transient transfection of a 3X Flag-hp50/dynamitin construct. Cells with a high level of 3X Flag-hp50 showed aberrant spindle morphology as previously described[7] and when treated with NDGA, hZW10 was observed to remain at the kinetochore. Cells on the same slide treated with NDGA but exhibiting low or no transfection with the 3X Flag- hp50/dynamitin construct showed accumulation of hZW10 at the poles (Figure 2B). This indicates that the NDGA induced accumulation of hZW10 at spindle poles is a dynein/dynactin dependent process.

### Kinetochore localization and k-MT attachments are required for hZW10 spindle pole localization

**Figure 4:**
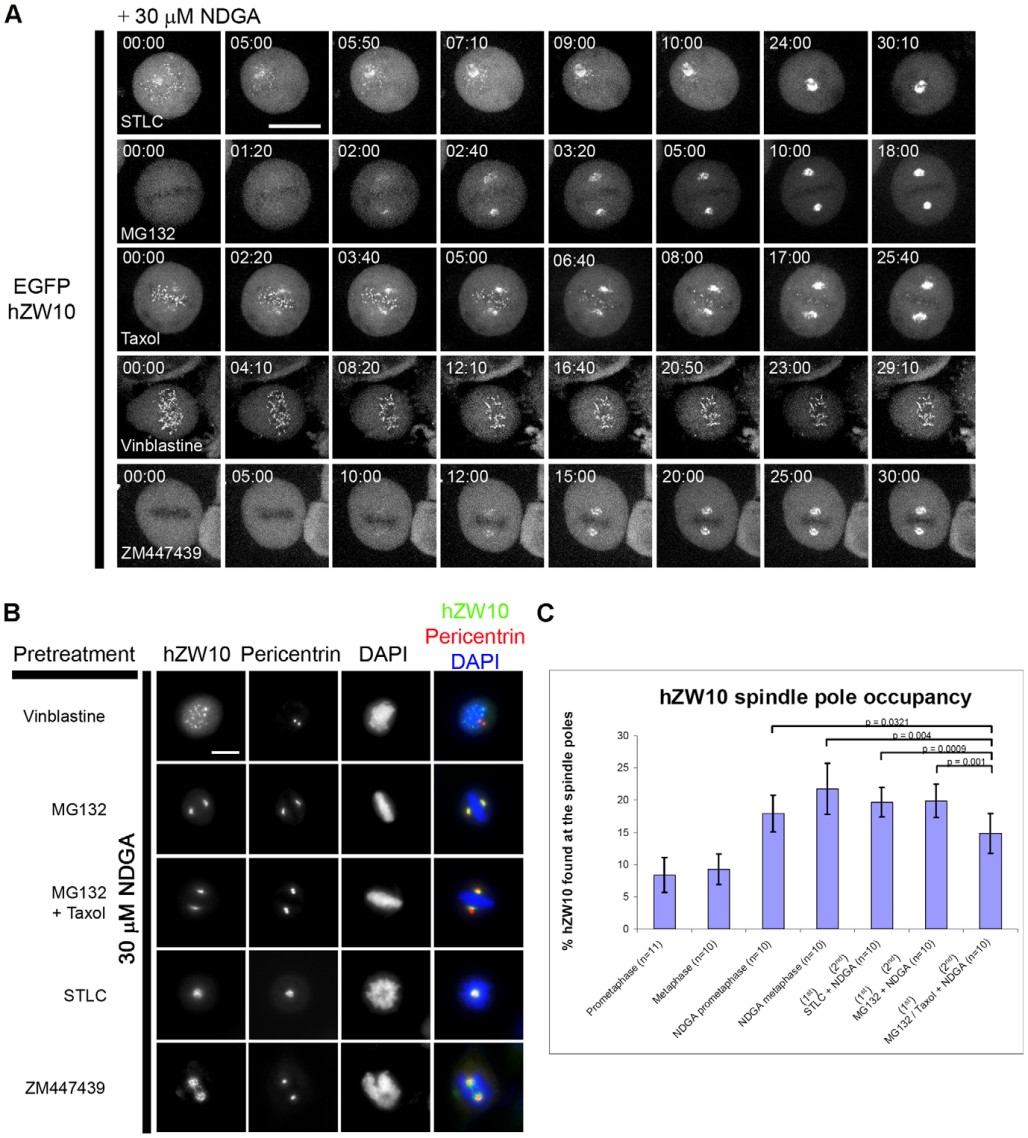
NDGA induced transport of hZW10 to spindle poles requires k-MT attachments. **A**) HeLa cells stably expressing EGFP-hZW10 were pre-treated with either: 7 µM STLC for 30 minutes, 12.5 µM MG132 for 1 hour, 12.5 µM MG132 for 1 hour followed by 12.5 µM MG132 and 1 µM taxol for 30 minutes, 0.5 µM vinblastine for 30 minutes or 2 µM ZM447439 for 30 minutes. 30 µM NDGA was added and the cells were immediately imaged using the spinning disk confocal microscope. Z-stacks of 1 µm thickness were collected every minute after NDGA treatment. Maximum projections of ∼20 Z-stacks are shown. The addition of NDGA induced EGFP-hZW10 transport to the spindle pole in all of the cells except those treated with vinblastine. Scale bar = 10 µm. **B**) HeLa cells pre-treated as above with either 0.5 µM vinblastine, 12.5 µM MG132, 12.5 µM MG132 + 1 µM taxol, 7 µM STLC or 2 µM ZM447439 were treated with 30 µM NDGA for 30 minutes and stained with hZW10 and pericentrin antibodies. hZW10 is observed to accumulate at spindle poles in all but the vinblastine treated cells. Chromosomes are stained with DAPI. Scale bar = 10 µm. **C**) Measurement of endogenous hZW10 intensity at spindle poles during mitosis and upon treatment with NDGA. (Error bars = +/− one standard deviation).

Having determined that NDGA can be used to study hZW10 at spindle poles in living cells, we next set out to analyze whether NDGA induced spindle pole accumulation of hZW10 was restricted to conditions when hZW10 was observed at the spindle pole without NDGA treatment (discussed above). EGFP-hZW10 expressing cells were pretreated with various inhibitors of spindle function followed by NDGA in live cells. Following NDGA addition EGFP-hZW10 readily accumulated at spindle poles in cells pre-treated with STLC, MG132, MG132 + taxol and ZM447439 (Aurora B kinase inhibitor) but not in those pre-treated with vinblastine (Figure 4A, 4B). hZW10 was not observed at the kinetochore or the spindle pole in MG132 or ZM447439 treated cells however the addition of NDGA resulted in EGFP-hZW10 spindle pole accumulation indicating that NDGA is able to induce spindle pole accumulation under conditions when hZW10 would not normally be observed at the kinetochore or spindle pole. Pretreatment with STLC or MG132 did not affect the dynamics or occupancy of hZW10 spindle pole accumulation from those observed for NDGA treated prometaphase and metaphase cells (Figure 4C). On the other hand, some EGFP-hZW10 still remained at kinetochores in MG132 + taxol pre-treated cells, even after 25 minutes of NDGA treatment (Figure 4A middle panel) and the spindle pole occupancy of hZW10 is reduced (Figure 4C). hZW10 localizes to both spindle poles and kinetochore during early and tensionless metaphase indicating it is not an artifact of NDGA treatment (Figure 1B, 1C). This suggests that inter-kinetochore tension, and or k-MT dynamics influence the ability of hZW10 to be released from kinetochores.

To determine whether NDGA itself had an affect on k-MT attachments or inter-kinetochore tension we examined cold stable k-MT attachments and observed no obvious changes in cells treated with NDGA (Figure S1A). NDGA treatment also had no effect on k-MT attachments as observed by electron microscopy (Figure S1B). Additionally, we found that tension, as measured by inter-kinetochore distance in MG132 treated cells, did not differ significantly upon 30 minute NDGA treatment (MG132: 1.80 +/− 0.21 µm, n = 76 vs MG132 + NDGA: 1.83 +/− 0.34 µm, n = 76).

We confirmed our live cell experiments by repeating the aforementioned treatments and analyzing the behavior of endogenous hZW10 by immunofluorescence staining (Figure 4B). When measuring hZW10 accumulation at the spindle poles, cells pre-treated with STLC or MG132 and then NDGA, exhibited up to 20% of total hZW10 accumulation at spindle poles. This constitutes a ∼2.35 fold increase when STLC pretreated cells are compared to prometaphase cells without NDGA treatment and a ∼2.15 fold increase for cells pretreated with MG132 compared to metaphase cells without NDGA treatment (Figure 4C). However, cells pre-treated with MG132 + taxol and then NDGA accumulated only ∼15% of total hZW10 at the spindle poles. This amounts to a ∼1.6 fold increase when compared to metaphase without NDGA treatment, and again suggests that inter-kinetochore tension may regulate hZW10 transport off kinetochores. These findings are in agreement with our previous studies showing that hZW10 kinetochore dynamics are regulated by bi-polar k-MT attachments and inter-kinetochore tension.[16], [17] Based upon our live cell and immunofluorescence results, we conclude that hZW10 spindle pole localization requires k-MT attachments and may also be regulated by inter-kinetochore tension.

**Figure 5:**
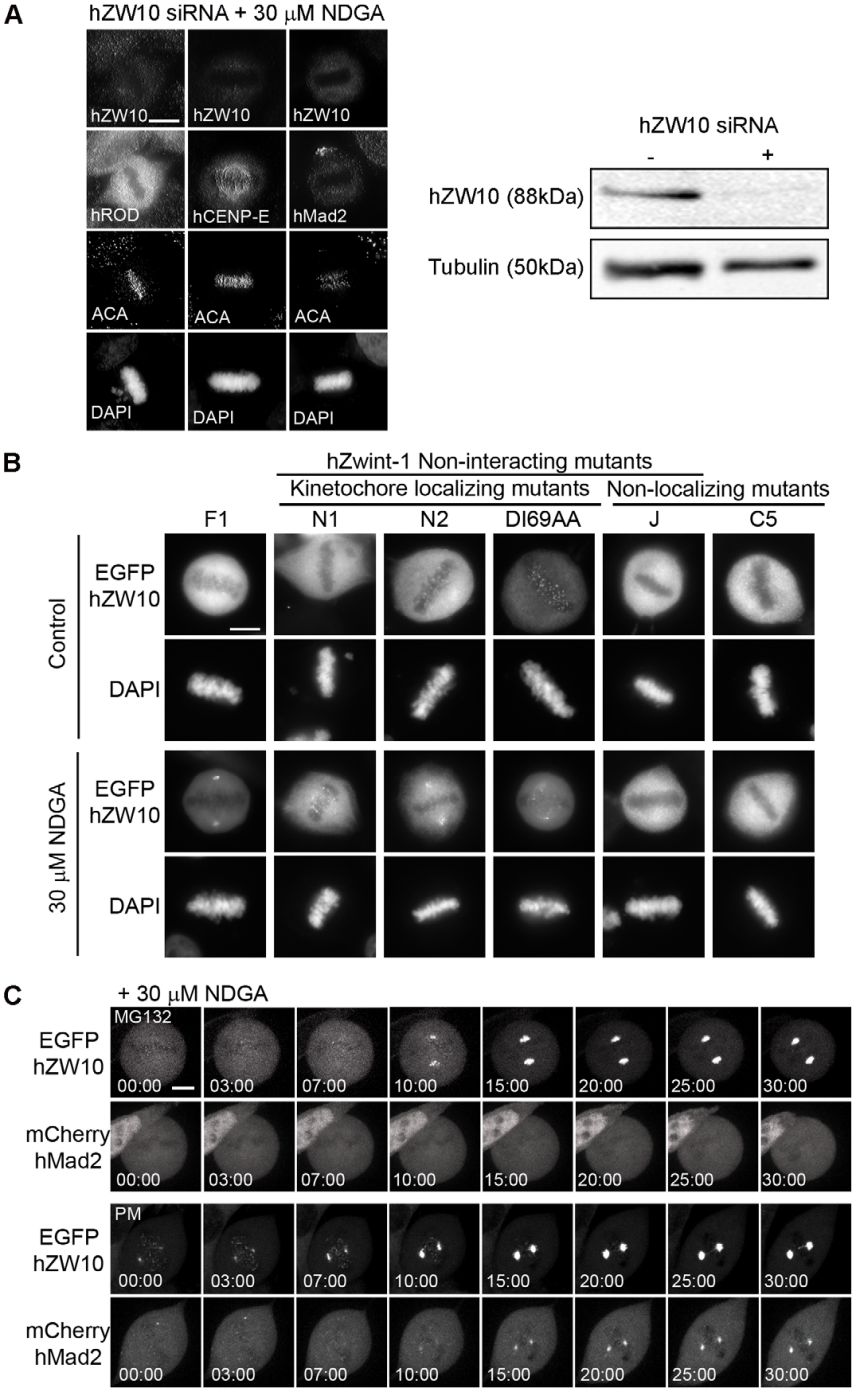
hZW10 is required for NDGA induced dynein/dynactin-mediated transport of kinetochore cargo. A) HeLa cells transfected with hZW10 siRNA for 72 hours and subsequently treated with 30 µM NDGA for 30 minutes were stained with hZW10, ACA and either hROD, CENP-E or hMad2 antibodies. Depletion of hZW10 prevented NDGA induced transport and accumulation of hROD, hCENP-E and hMad2 onto spindle poles. Scale bar = 10 µm. On the right is an immunoblot of HeLa cell lysates depleted of hZW10 after 72 hours of siRNA and stained with the corresponding antibodies. B) HeLa cells transiently transfected with EGFP-hZW10 wild type or mutants for 24 hours and then treated with 30 µM NDGA for 30 minutes. Mutants C5 (1–410aa) and J (L^248^LRPQL) are unable to localize to the kinetochore and do not accumulate at the spindle pole following NDGA treatment. Mutants N1 (52–779aa), N2 (75–779aa) and DI69AA are able to localize to the kinetochore but do not interact with hZwint-1 and are able to transport to the pole but do not accumulate to wild type levels following NDGA treatment. Chromosomes are stained with DAPI. Scale bar = 10 µm. C) Prometaphase or MG132 treated (12.5 µM for 1 hour) HeLa cells stably expressing EGFP-hZW10 and transiently transfected with mCherry-hMad2 were imaged live upon treatment with 30 µM NDGA. Only EGFP-hZW10 is observed to transport onto spindle poles in the MG132 arrested cells, while both EGFP-hZW10 and mCherry-hMad2 transport onto spindle poles in prometaphase cells. Time is indicated in minutes:seconds. Scale bar = 10 µm.

In our previous studies we generated and characterized a collection of hZW10 mutants that either no longer localize to kinetochores or are unable to interact with hZwint-1.[17] To test whether kinetochore localization is necessary for spindle pole localization we subjected cells transfected with one of the kinetochore non-localization mutants (insertion mutant J: insertion of LRPQL at amino acid 248; or truncation C5: 1–410 amino acids) to NDGA treatment. Fluorescence microscopy revealed that in the presence of NDGA, EGFP-hZW10 J and EGFP-hZW10 C5 did not accumulate at the spindle poles (Figure 5B). Therefore, we deduce that hZW10 spindle pole localization requires kinetochore localization prior to transport. The dynamics of hZW10 are regulated by tension as well as by interaction with hZwint-1. In our previous study we found that a subset of hZW10 mutants which were unable to interact with hZwint-1 were still able to localize to the kinetochore. Further analysis of truncation N1 (52–779 amino acids) revealed that although it was able to localize to the kinetochore with the same timing as the wild type protein it had altered FRAP dynamics and impaired checkpoint activity.[17] As such we examined cells transfected with one of our hZwint-1 non-interacting mutants which are able to localize to the kinetochore (truncation N1: 52–779 amino acids; truncation N2: 75–779 amino acids; or site-directed mutant: DI69AA) for their ability to accumulate at the spindle pole following NDGA treatment. Fluorescence microscopy revealed that in the presence of NDGA, EGFP-hZW10 N1, EGFP-hZW10 N2 and EGFP-hZW10 DI69AA were able to localize to the spindle pole but had reduced accumulation at the spindle poles compared to wild-type hZW10 (Fl: 1–779 amino acids) and retained visible kinetochore staining (Figure 5B). This indicates that these mutants are able to be transported but may not stably accumulate at the poles.

### NDGA induces the transport of RZZ complex components but not the scaffold protein hZwint-1

**Figure 6:**
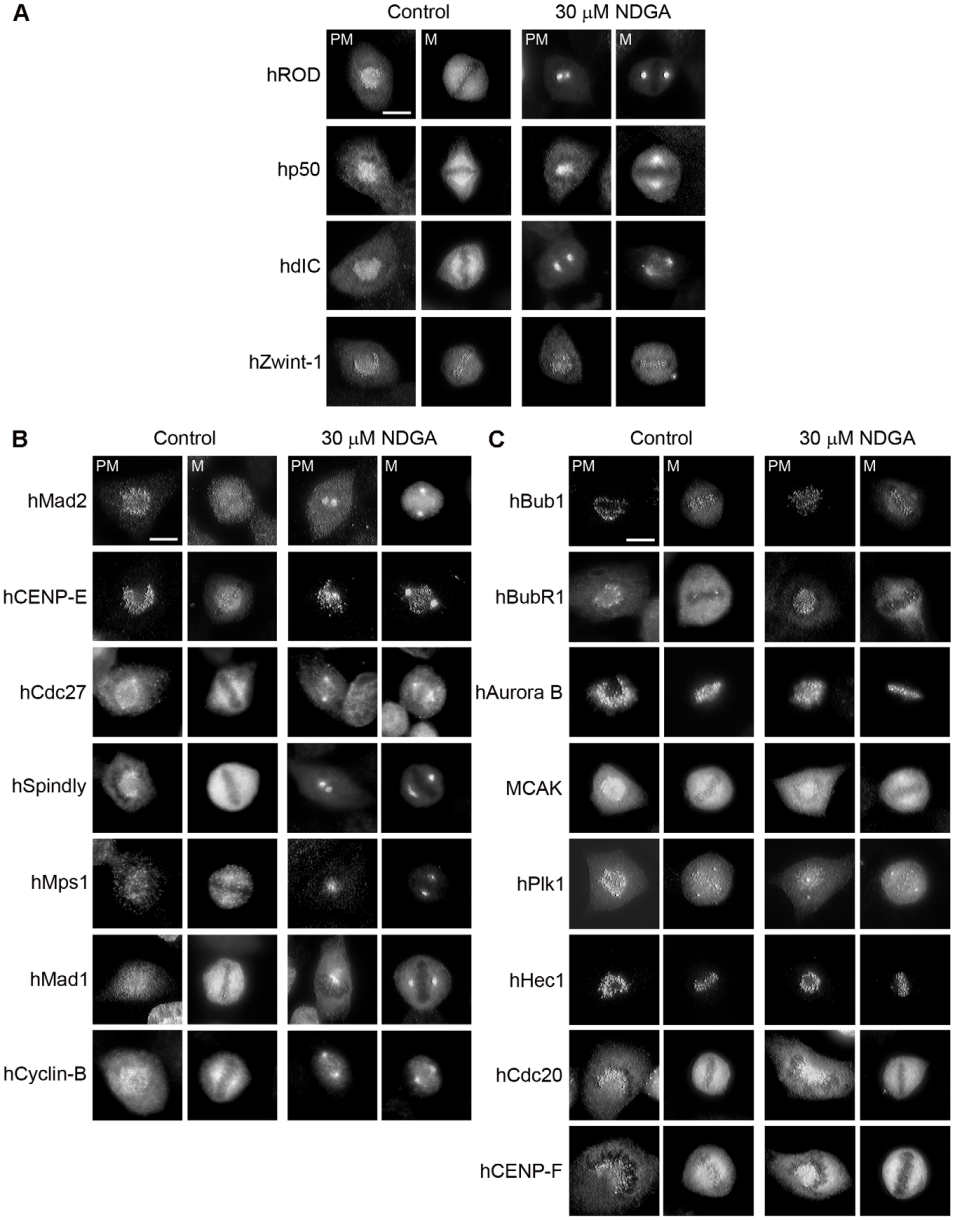
NDGA induced transport of kinetochore cargo onto spindle poles. **A–C**) HeLa cells treated with 30 µM NDGA for 30 minutes and stained with the indicated antibodies. Panel A depicts the RZZ component hROD, the dynein/dynactin components hp50 and hdIC and the hZW10 interactor hZwint-1. Panel B depicts non-RZZ complex kinetochore components transported by NDGA treatment while panel C depicts kinetochore and centromere components not transported by NDGA treatment. Co-staining with DAPI, hZW10 and ACA for each experiment is shown in Figures S2–S5. Scale bar = 10 µm.

hZW10 is part of the evolutionarily conserved RZZ complex, which includes hZW10, hROD and hZwilch.[14], [20] To date, the function of the complex has been shown to be interdependent on all of its components.[13], [18], [29] We therefore analyzed whether hROD behaved similarly to hZW10 upon treatment with NDGA. Immunofluorescence staining of hROD at spindle poles upon NDGA treatment suggests that the entire RZZ complex accumulates at spindle poles in the presence of NDGA (Figure 6A, S2). We confirmed this by examining hZwilch and found it also accumulates at spindle poles following NDGA treatment (data not shown). In addition to the RZZ components, hZW10 is also known to interact with hZwint-1 and dynamitin (hp50).[30], [31] We therefore analyzed whether hZwint-1 and hp50 behave similarly to hZW10 in response to NDGA treatment. Our results show that hp50 but not hZwint-1 accumulates at spindle poles in the presence of NDGA (Figure 6A, S2). Additionally, we also determined the localization of dynein intermediate chain (hdIC) in the presence of NDGA and found that it also accumulated at spindle poles (Figure 6A, S2). Since dynein/dynactin mediated transport is the only mechanism known to be responsible for RZZ spindle pole accumulation,[12] our data suggests that NDGA induces the accumulation of the entire RZZ complex at the spindle poles through dynein/dynactin mediated transport.

### Dynein/dynactin transports a subset of mitotic checkpoint proteins from kinetochores to spindle poles

There are several mitotic checkpoint proteins, that similar to hZW10 and hROD, are thought to be transported from kinetochores to spindle poles via dynein/dynactin.[7], [32] Reasoning that bona fide dynein/dynactin cargoes would be expected to accumulate at spindle poles upon exposure to NDGA we screened an array of mitotic checkpoint proteins for their response to NDGA. We first examined hBubR1, hBub1, hCENP-E and hMad2, of which, hBubR1, CENP-E and hMad2 have previously been implicated as cargo of dynein/dynactin dependent transport.[7] Upon treatment with NDGA, we observed that hCENP-E and hMad2 accumulated at the spindle poles, while hBubR1 and hBub1 did not (Figure 6B, 6C, S3). After 30 minutes of NDGA treatment, hMad2 accumulation at spindle poles mirrored that of hZW10 in prometaphase (Figure 5C). The immunofluorescence results were confirmed by time-lapse fluorescence microscopy of HeLa cells transiently transfected with the corresponding YFP and GFP fusion constructs (Figure S6). When we extended our NDGA assay to test for the transport of other mitotic checkpoint proteins off kinetochores, we found that hMad1, hMps1, hSpindly, hCdc27 and cyclin-B were also transported by dynein/dynactin to the spindle poles (Figure 6B, S4). We also found kinetochore proteins that were insensitive to NDGA induced transport. These included: ACA (anti-centromere antigen: CENP-A, B and C), hCENP-F, hAurora-B, hCdc20, hMCAK, hPlk1 and hHec1 (Figure 6C, S5).

To show that the NDGA induced transport of checkpoint proteins was indeed dynein/dynactin dependent, we depleted cells of hZW10 and thus disrupted the recruitment of dynein/dynactin to the kinetochores.[33] In hZW10 depleted cells, neither hROD, hMad2 nor hCENP-E were not found at spindle poles following NDGA treatment (Figure 5A). Furthermore, in HeLa cells co-expressing EGFP-hZW10 and mCherry-hMad2, NDGA selectively transported mCherry-hMad2 to spindle poles in prometaphase but not MG132 arrested cells (Figure 5C). This indicates that NDGA does not interfere with k-MT attachment and does not result in the re-recruitment of hMad2 to kinetochores.

Our use of NDGA to characterize dynein/dynactin mitotic cargo is an extension of previous work from Ted Salmon's lab which identified several dynein/dynactin cargo through the use of ATP depletion.[7] This current study confirmed that hMad2, and hCENP-E are dynein/dynactin cargo and identified hSpindly, hCdc27, cyclin-B, the RZZ complex and the hMps1 kinase as additional cargo. Moreover, we were also able to categorize several kinetochore components which are not dynein/dynactin cargo including hHec1, hMCAK, hAuroraB, hBubR1, hBub1, hZwint-1, hPlk1, hCENP-F and hCdc20.

## Discussion

During mitosis hZW10 localizes to kinetochores from late prophase through early metaphase and to the spindle pole in late prometaphase and early metaphase (Figure 1A, 1B). Our previous work indicated that hZW10 is a dynamic component of metaphase kinetochores[17] and here, using FRAP, we show that hZW10 is also a dynamic component of spindle poles in both prometaphase and metaphase (Figure 1D). While kinetochore localization of hZW10 is independent of k-MTs, spindle pole localization depends on the establishment of k-MT attachment. This is most apparent when examining hZW10 localization upon treatment with the MT depolymerizing compound vinblastine, where hZW10 is clearly localized to kinetochores but completely absent from spindle poles. Conversely, treatment with STLC or MG132 + low-dose taxol did not affect hZW10 spindle pole localization (Figure 1C).

A previously published study indicated that hZW10 spindle pole localization could be enhanced by treatment with the lipoxygenase inhibitor NDGA.[11] Indeed, treatment with NDGA resulted in spindle pole accumulation of hZW10 to higher levels than normally observed (Figure 2A, 2C). Furthermore, FRAP of spindle pole associated EGFP-hZW10 indicated that in the presence of NDGA EGFP-hZW10 becomes completely stable at spindle poles (Figure 2D). Interestingly, NDGA also induced hZW10 spindle pole accumulation in late metaphase, a time when hZW10 is clearly absent from kinetochores and spindle poles in control cells. The ability of NDGA to induce spindle pole accumulation of hZW10 in late metaphase implies that hZW10 kinetochore and spindle pole levels are extremely low and/or highly dynamic during this stage of mitosis. Furthermore, it implies that hZW10 is still being transported onto the spindle pole even though the mitotic checkpoint has been silenced.

NDGA may be affecting the ability of the RZZ complex to dissociate from the spindle pole, perhaps by directly stabilizing its interaction with dynein/dynactin. Interestingly, our NDGA results are similar to those of ATP depletion studies where mitotic checkpoint proteins are also observed to accumulate at spindle poles.[7], [32], [34] NDGA has been shown to reduce cellular ATP levels to 40% of control cells 30 minutes after treatment[35] and might therefore affect ATP-dependent processes involved in the release of checkpoint proteins from spindle poles. We do not believe this is the case as the addition of an ATP regeneration system did not prevent NDGA induced hZW10 accumulation at centrosomes in interphase cells.[11] Another possibility is that NDGA may be affecting the modification or stabilization of MTs. NDGA has been shown to protect MTs from depolymerizing agents such as vinblastine[36] and preliminary computational modeling experiments show direct binding of NDGA to MTs (personal communication with Dr. J. Tuszynski, University of Alberta). This stabilization/modification of MTs may prevent release of dynein/dynactin and its cargos from the spindle pole resulting in the stable association observed for EGFP-hZW10.

To address the mechanism involved in NGDA-induced spindle pole accumulation of hZW10, we analyzed hZW10 mutants[17] that are unable to localize to kinetochores and observed no spindle pole accumulation (Figure 5B). We also found that hZW10 mutants which were able to localize to the kinetochore but unable to interact with hZwint-1 had altered spindle pole accumulation. The hZwint-1 non-interacting mutant N1 has been previously shown to be dynamic at prometaphase kinetochores compared with stable wild type hZW10.[17] Because N1 is more dynamic at prometaphase we expected it would have faster and greater accumulation at spindle poles following NDGA treatment, however, we actually observed reduced spindle pole accumulation. The truncated protein is still transported to the spindle pole but the reduced accumulation may indicate that it is not stably bound at the pole. While this mutant is unable to interact with hZwint-1 it is unlikely that hZwint-1 is involved in stable attachment of hZW10 at the spindle pole as hZwint-1 does not localize there. The domain of hZW10 required for hZwint-1 interaction may therefore be involved in binding at the spindle pole. Alternatively, hZW10 interaction with hZwint-1 may prime hZW10 for transport to the spindle pole or its retention there.

Arasaki et al. showed that NDGA induced centrosomal accumulation of hZW10 in interphase is dependent on dynein/dynactin.[11] Our study confirmed that NDGA induced accumulation of hZW10 at the spindle poles in mitosis is dynein/dynactin dependent. Disrupting dynein/dynactin function by hp50/dynamitin overexpression[28] completely abolished hZW10 accumulation at spindle poles following NDGA treatment (Figure 2B). Furthermore, inhibiting dynein/dynactin recruitment to the kinetochore with siRNA-mediated hZW10 depletion[33] prevented NDGA-induced spindle pole accumulation of hMad2, hROD or hCENP-E (Figure 5A). Pre-treating cells with vinblastine, to depolymerize all k-MTs, also prevented accumulation of hZW10 at the poles of NDGA treated cells (Figure 4A, 4B), thus confirming that kinetochore proteins are transported to the poles by dynein/dynactin mediated transport along k-MTs and not directly recruited to the spindle poles. This was also observed directly by live cell imaging of streaming of EGFP-hZW10 to the spindle poles following NDGA treatment (Figure 3, Movie S2).

Using NDGA we have been able to identify a subset of kinetochore proteins, in addition to hZW10, that are transported off kinetochores by dynein/dynactin as well as those that are not (Figure 6). As expected the dynein/dynactin components hdIC and hp50/dynamitin accumulated at the spindle poles in NDGA treated cells. We also found that the RZZ complex (hZW10, hROD and hZwilch) and its newly identified partner hSpindly also accumulated at spindle poles following NDGA treatment. In addition to the RZZ complex, we also observed hMad1, hMad2 and hCENP-E spindle pole accumulation after NDGA treatment. Our findings confirm previous studies showing the streaming of hMad2 along k-MTs[26] as well as observed spindle pole localization of Mad1,[37] and ATP depletion studies showing spindle pole accumulation of Mad2 and CENP-E.[7] Additionally, we found that hMps1, hCdc27 and cyclin-B also accumulate at spindle poles in the presence of NDGA. Surprisingly, NDGA did not induce spindle pole accumulation of hCdc20, which is observed at spindle poles in mitosis,[38], [39] or hBubR1, which has been shown to accumulate at spindle poles after ATP depletion.[7] It is unclear whether this discrepancy is due to differences in experimental methods or systems. While Rod and Mad2 are known to stream from kinetochores to the poles, BubR1 does not normally shed from kinetochores.[40] In addition, the normal kinetochore localization timing of Cdc20 and BubR1 is distinct from that of Mad2 and the RZZ complex. While Mad2 and ZW10 vacate the kinetochore upon MT attachment and bipolar alignment respectively,[16], [26], [41] BubR1 and Cdc20 remain at the kinetochore at metaphase and into anaphase respectively indicating that their removal is not required for inactivation of the checkpoint.[38], [40], [42], [43] The separation of Mad2 from BubR1 and Cdc20 at metaphase implicates the possible disassembly of the MCC complex for checkpoint silencing. Furthermore, the precise mechanism of NDGA induced spindle pole accumulation is unknown and hCdc20 and hBubR1 may be transiently transported by dynein/dynactin but not retained at spindle poles. On the other hand, the majority of the remaining NDGA insensitive proteins (hZwint-1, hHec1, hBub1, hPlk1 and hAurora B kinase) are known to be stable kinetochore components or have only partial recovery as examined by FRAP[37], [38], [44], [45], [46], [47] and would not be expected to be dynein/dynactin cargo.

Within the pool of NDGA responsive kinetochore proteins there appears to be two distinct types of proteins; those that only accumulate at the spindle poles following NDGA treatment when the checkpoint is active (e.g. Mad2) and those that accumulate throughout mitosis (e.g. hZW10). We observed that hZW10 is able to accumulate at spindle poles from prometaphase through anaphase and when cells are in a checkpoint inactive state with complete chromosome alignment, as achieved by MG132 treatment. In contrast, Mad2 accumulated in prometaphase cells but not in MG132 treated cells (Figure 5C). These observed differences may be due to a difference in kinetochore recruitment or a change in release from spindle poles following checkpoint silencing. The observed timing of accumulation for hZW10 may be a reflection of its role in recruiting dynein/dynactin to the kinetochore. In addition, hZW10 may be required for regulating the removal of checkpoint proteins from the kinetochore so that the checkpoint is not erroneously turned on or off. The slowed and reduced NDGA induced accumulation of hZW10 at the poles when cells have fully aligned chromosomes but lack inter-kinetochore tension (MG132 and taxol) may be indicative of this potential regulatory role (Figure 4C). Future studies may shed light upon the role of hZW10 in checkpoint regulation.

## Materials and Methods

### Cell Culture

HeLa cells were obtained from ATCC (CCL-2) and grown in DMEM with 10% FCS and 2 mM L-Glutamine at 37°C in 5% CO_2_. For FRAP experiments, HeLa cell media was supplemented with 1 M HEPES buffer (pH 7.4, Gibco) to a final concentration of 7 mM. Vinblastine (Sigma) was used at a final concentration of 0.5 µM unless otherwise stated. STLC (Sigma) was used at a final concentration of 2 µM. NDGA (Biomol) was used at a final concentration of 30 µM. MG132 (VWR) was used at a final concentration of 12.5 µM. Taxol (Sigma) was used at a final concentration of 1 µM. ZM447439 (Astra) was used at a final concentration of 2 µM. NDGA, STLC, ZM447439 and vinblastine treatments were for 30 minutes, MG132 was 1 hour and MG132 with taxol was 1 hour MG132 followed by 30 minutes with MG132 and taxol together.

### Western Blotting

HeLa cells were harvested for western blotting as previously described[48] and western blots were stained and analyzed as previously described.[17]

### Fluorescence microscopy

HeLa cells were processed for immunofluorescence as previously described.[17]

hZW10 was visualized using rabbit or rat anti-hZW10 antibodies at a 1/1500 dilution and 1/500 dilution respectively[13] and ACA was visualized using human ACA sera at a 1/3000 dilution (gift from Dr. Marvin Fritzler, University of Calgary). Pericentrin was visualized using rabbit-anti pericentrin antibodies (Abcam) at 1/1000 dilution. hROD was visualized using rabbit anti-hROD antibodies[13] at a 1/1000 dilution. hCENP-E was visualized using rabbit anti-hCENP-E antibodies[48] at a dilution of 1/1000. hMad2 was visualized using rabbit anti-hMad2 antibodies (gift from Dr. Salmon) at a dilution of 1/250. hBubR1 was visualized using rabbit anti-hBubR1 antibodies[49] at a dilution of 1/500. hp50 was visualized using mouse anti-hp50 antibodies (gift from Dr. Valle) at a dilution of 1/750. hdIC was visualized using the 74.1 mouse antibody (Abcam) at a dilution of 1/500. hZwint-1 was visualized using rat anti-hZwint-1 antibodies (unpublished) at a dilution of 1/1000. Cyclin B was visualized with rabbit anti-cyclin B antibodies at a dilution of 1/250 (Santa Cruz). hBub1 was visualized using rat anti-hBub1 antibodies[50] at a dilution of 1/1000. hMps1 was visualized using rabbit anti-hMps1 antibodies[51] at a dilution of 1/1000. hMad1 was visualized using mouse anti-hMad1 antibodies[52] at a dilution of 1/500. Tubulin was visualized using the B512 mouse anti-Tubulin antibody (Sigma) at a dilution of 1/1500. hCENP-F was visualized using rabbit anti-hCENP-F antibodies[48] at a dilution of 1/1000. hAurora B was visualized using rabbit anti-hAurora B antibodies (Abcam) at a dilution of 1/1500. hCdc20 was visualized using rabbit anti-hCdc20 antibodies[53] at a dilution of 1/500. MCAK was visualized using rabbit anti-MCAK (Abcam) antibodies at a dilution of 1/500. hPlk1 was visualised using mouse anti-hPlk1 antibodies (gift from Dr. Lee) at a dilution of 1/1000. hHec1 was visualized using rabbit anti-hHec1 antibodies (Abcam) at a dilution of 1/1500. All secondary antibodies conjugated to Alexa 488, 555 or 647 were used at a dilution of 1/1000 (Molecular Probes). Cold stable MTs were generated by incubation of cells with ice cold media and on ice for 10 minutes. A Zeiss AxioPlan2 microscope equipped with epifluorescence optics was used to collect the images. Cells were visualized with a 100X Plan-Apochromatic objective (NA1.4) and images were captured with a Photometrics CoolSNAP HQ CCD camera (Roper Scientific Inc., Trenton, NJ) that was controlled with a personal computer running Metamorph software (v7.1, Universal Imaging Corporation, Downingtown, PA). The coverslips were mounted using Mowoil mounting media (Calbiochem) and imaging was performed at room temperature. Image processing was performed using Photoshop 7.0 (Adobe Systems Inc., Mountain View CA).

Spindle pole or total kinetochore intensity was measured using Imaris software (Bitplane Scientific Software) from Z-stacks collected as previously described. [16] Spindle pole intensity was measured by outlining the spindle pole, calculating its fluorescence intensity in 3D and then comparing the value to the total fluorescence intensity of the entire cell. In cells with two visible spindle poles, both poles were measured individually and added together before comparing to the total cell intensity. Statistics and graphing were preformed using Excel (Microsoft).

### Electron Microscopy

For Electron Microscopy, cells were fixed in 3% glutaraldehyde in Millonig's phosphate buffer for 1 hr at room temperature. Post-fixation was in 2% OsO_4_ for 20 minutes. The cells were dehydrated in ethanol, and then infiltrated with Polybed 812 resin (Polysciences). Polymerization was performed at 37°C for 24 hrs. Silver-gray sections were cut with an ultramicrotome (Leica) equipped with a diamond knife, and sections were stained with uranyl acetate and lead citrate and examined in a electron microscope (H-7000:Hitachi).

#### siRNA

hZW10 siRNA knock-down was performed as previously described.[17]

### Transient Transfection and Permanent Cell Line Selection

HeLa cells grown to 60% confluence on coverslips in 35 mm dishes were transiently transfected with 2 µg of the EGFP constructs or 4 µg of the 3X Flag-hp50 construct with 10 µl 1 mg/ml linear Polyethylenimine, MW ∼25,000 (Cedarlane) for 24 hours. Selection of the permanent cell line expressing EGFP-hZW10 as previously described.[17]

### Live Cell Imaging

FRAP was performed as previously described.[17] Briefly, single spindle poles were laser ablated with 10 laser pulses and the subsequent fluorescence recovery was observed for 90–120 seconds at 1–10 seconds intervals. Data was collected using the Zeiss LSM software (Zeiss), processed using Excel (Microsoft) and graphed using Prism software. Live cell imaging was performed as previously described.[16] The data was analyzed using UltraVIEW ERS software (PerkinElmer).

## Supporting Information

Figure S1 **NDGA treatment does not disrupt k-MT attachments.** **A**) HeLa cells arrested with 12.5 µM MG132 and subsequently treated with NDGA were exposed to ice cold media for 10 minutes and harvested, immunofluorescence stained and imaged in 3D using confocal microscopy. Tubulin and ACA staining shows that NDGA does not affect k-MT attachments. Insets show enlargements of k-MT attachments. Tubulin is shown in green, ACA in red. Scale bar = 10 µm. ∼50 kinetochores per cell, as observed by ACA staining, were analyzed for MT attachments and scored as ratio of attached/total. MG132 n = 3 cells, 164 kinetochores and NDGA n = 10 cells, 490 kinetochores. Error bars = +/− one standard deviation. **B**) HeLa cells treated with NDGA for 0.5 hours (top), 3 hours (middle) or 6 hours (bottom) were fixed and analyzed for k-MT attachments using electron microscopy. Shown are three different magnifications of chromosomes and their corresponding k-MTs. In all three NDGA treatments normal kinetochore plates as well as k-MTs are observed. Yellow arrows indicate kinetochores while the arrow heads indicate k-MTs.
(TIF)

Figure S2 **NDGA transport of hZW10, hROD, hdIC and hp50.** **A-D**) HeLa cells treated with 30 µM NDGA for 30 minutes and stained with hZW10, ACA and either: hROD (A), hZwint-1 (B), hp50 (C) or hdIC (D) antibodies. hZW10, hROD, hdIC and hp50 are observed to accumulate at spindle poles while hZwint-1 does not. Chromosomes are stained with DAPI. Scale bar = 10 µm.
(TIF)

Figure S3 **hMad2 and hCENP-E but not hBub1 or hBubR1 are transported to spindle poles in the presence of NDGA.** **A–D**) HeLa cells treated with 30 µM NDGA for 30 minutes and stained with hZW10, ACA and either: hMad2 (A), hBubR1 (B), hBub1 (C) or hCENP-E (D) antibodies. hZW10, hCENP-E and hMad2 are observed to accumulate at spindle poles while hBub1 and hBubR1 do not. Chromosomes are stained with DAPI. Scale bar = 10 µm.
(TIF)

Figure S4 **hMps1, hSpindly, hMad1, Cdc27 and cyclin- B are transported to spindle poles in the presence of NDGA.** **A–E**) HeLa cells treated with 30 µM NDGA for 30 minutes and stained with hZW10, ACA and either: hMps1 (A), hSpindly (B), hMad1 (C), hCdc27 (D) or cyclin-B (E) antibodies. hZW10, hMps1, hMad1, hCdc27, cyclin-B and hSpindly are observed to accumulate at spindle poles. Chromosomes are stained with DAPI. Scale bar = 10 µm.
(TIF)

Figure S5 **hAurora B, MCAK, hPlk1, hHec1, hCENP-F and hCdc20 are not transported to spindle poles in the presence of NDGA.** **A–F**) HeLa cells treated with 30 µM NDGA for 30 minutes and stained with hZW10, ACA and either: hAurora B (A), MCAK (B), hPlk1 (C), hHec1 (D), hCENP-F (E) or hCdc20 (F) antibodies. Only hZW10 is observed to accumulate at spindle poles. Chromosomes are stained with DAPI. Scale bar = 10 µm.
(TIF)

Figure S6 **Live cell analysis of hp50, hBub1, cyclin- B and hMad2 response to NDGA treatment.** HeLa cells transiently transfected with either: YFP-hp50, EGFP-hBub1, cyclin-B-GFP or EGFP-hMad2 were imaged using a spinning disk confocal microscope. Upon addition of NDGA, the cells were imaged every 1 minute as a Z-stack of ∼20 images 1 µm apart. Maximum projections are shown. YFP-hp50, cyclin-B-GFP and EGFP-hMad2 are observed to accumulate at spindle poles upon NDGA treatment while EGFP-hBub1 does not. Time is indicated as minutes:seconds. Scale bar = 10 µm.
(TIF)

Movie S1 **NDGA induced accumulation of EGFP-hZW10.** HeLa cells stably expressing EGFP-hZW10 were pre-treated with MG132 and then with 30 µM NDGA and immediately imaged using the spinning disk confocal microscope. Maximum projections of ∼20 1 µm Z-stacks taken 10 seconds apart are shown. EGFP-hZW10 is seen to accumulate at the spindle pole within minutes of adding NDGA. Time shown as minutes:seconds.milliseconds.
(MOV)

Movie S2 **NDGA induced accumulation of EGFP-hZW10 by kinetochore ‘shedding’.** HeLa cells stably expressing EGFP-hZW10 were treated with 30 µM NDGA and imaged using the spinning disk confocal microscope. Maximum projections of 5 1 µm Z-stacks taken 2.26 seconds apart are shown. EGFP-hZW10 is seen accumulated at the spindle poles at the start of the movie and EGFP-hZW10 foci are seen steaming towards the spindle pole. Time shown as minutes:seconds.milliseconds. Scale bar = 8 µm.
(MOV)

## References

[1] Kops GJ, Weaver BA, Cleveland DW (2005). On the road to cancer: aneuploidy and the mitotic checkpoint.. Nat Rev Cancer.

[2] Chan GK, Liu ST, Yen TJ (2005). Kinetochore structure and function.. Trends Cell Biol.

[3] Musacchio A, Salmon ED (2007). The spindle-assembly checkpoint in space and time.. Nat Rev Mol Cell Biol.

[4] Cheeseman IM, Desai A (2008). Molecular architecture of the kinetochore-microtubule interface.. Nat Rev Mol Cell Biol.

[5] King RW, Peters JM, Tugendreich S, Rolfe M, Hieter P (1995). A 20S complex containing CDC27 and CDC16 catalyzes the mitosis-specific conjugation of ubiquitin to cyclin B.. Cell.

[6] Sudakin V, Ganoth D, Dahan A, Heller H, Hershko J (1995). The cyclosome, a large complex containing cyclin-selective ubiquitin ligase activity, targets cyclins for destruction at the end of mitosis.. Mol Biol Cell.

[7] Howell BJ, McEwen BF, Canman JC, Hoffman DB, Farrar EM (2001). Cytoplasmic dynein/dynactin drives kinetochore protein transport to the spindle poles and has a role in mitotic spindle checkpoint inactivation.. J Cell Biol.

[8] Ban KH, Torres JZ, Miller JJ, Mikhailov A, Nachury MV (2007). The END network couples spindle pole assembly to inhibition of the anaphase-promoting complex/cyclosome in early mitosis.. Dev Cell.

[9] Clute P, Pines J (1999). Temporal and spatial control of cyclin B1 destruction in metaphase.. Nat Cell Biol.

[10] Kraft C, Herzog F, Gieffers C, Mechtler K, Hagting A (2003). Mitotic regulation of the human anaphase-promoting complex by phosphorylation.. EMBO J.

[11] Arasaki K, Tani K, Yoshimori T, Stephens DJ, Tagaya M (2007). Nordihydroguaiaretic acid affects multiple dynein-dynactin functions in interphase and mitotic cells.. Mol Pharmacol.

[12] Basto R, Scaerou F, Mische S, Wojcik E, Lefebvre C (2004). In vivo dynamics of the rough deal checkpoint protein during Drosophila mitosis.. Curr Biol.

[13] Chan GK, Jablonski SA, Starr DA, Goldberg ML, Yen TJ (2000). Human Zw10 and ROD are mitotic checkpoint proteins that bind to kinetochores.. Nat Cell Biol.

[14] Karess R (2005). Rod-Zw10-Zwilch: a key player in the spindle checkpoint.. Trends Cell Biol.

[15] Wojcik E, Basto R, Serr M, Scaerou F, Karess R (2001). Kinetochore dynein: its dynamics and role in the transport of the Rough deal checkpoint protein.. Nat Cell Biol.

[16] Famulski JK, Chan GK (2007). Aurora B kinase-dependent recruitment of hZW10 and hROD to tensionless kinetochores.. Curr Biol.

[17] Famulski JK, Vos L, Sun X, Chan G (2008). Stable hZW10 kinetochore residency, mediated by hZwint-1 interaction, is essential for the mitotic checkpoint.. J Cell Biol.

[18] Basto R, Gomes R, Karess RE (2000). Rough deal and Zw10 are required for the metaphase checkpoint in Drosophila.. Nat Cell Biol.

[19] Williams BC, Goldberg ML (1994). Determinants of Drosophila zw10 protein localization and function.. J Cell Sci.

[20] Scaerou F, Starr DA, Piano F, Papoulas O, Karess RE (2001). The ZW10 and Rough Deal checkpoint proteins function together in a large, evolutionarily conserved complex targeted to the kinetochore.. J Cell Sci.

[21] Williams BC, Karr TL, Montgomery JM, Goldberg ML (1992). The Drosophila l(1)zw10 gene product, required for accurate mitotic chromosome segregation, is redistributed at anaphase onset.. J Cell Biol.

[22] Wendell KL, Wilson L, Jordan MA (1993). Mitotic block in HeLa cells by vinblastine: ultrastructural changes in kinetochore-microtubule attachment and in centrosomes.. J Cell Sci.

[23] Skoufias DA, DeBonis S, Saoudi Y, Lebeau L, Crevel I (2006). S-trityl-L-cysteine is a reversible, tight binding inhibitor of the human kinesin Eg5 that specifically blocks mitotic progression.. J Biol Chem.

[24] Rock KL, Gramm C, Rothstein L, Clark K, Stein R (1994). Inhibitors of the proteasome block the degradation of most cell proteins and the generation of peptides presented on MHC class I molecules.. Cell.

[25] Mallik R, Petrov D, Lex SA, King SJ, Gross SP (2005). Building complexity: an in vitro study of cytoplasmic dynein with in vivo implications.. Curr Biol.

[26] Howell BJ, Hoffman DB, Fang G, Murray AW, Salmon ED (2000). Visualization of Mad2 dynamics at kinetochores, along spindle fibers, and at spindle poles in living cells.. J Cell Biol.

[27] Merdes A, Heald R, Samejima K, Earnshaw WC, Cleveland DW (2000). Formation of spindle poles by dynein/dynactin-dependent transport of NuMA.. J Cell Biol.

[28] Echeverri CJ, Paschal BM, Vaughan KT, Vallee RB (1996). Molecular characterization of the 50-kD subunit of dynactin reveals function for the complex in chromosome alignment and spindle organization during mitosis.. J Cell Biol.

[29] Williams BC, Li Z, Liu S, Williams EV, Leung G (2003). Zwilch, a new component of the ZW10/ROD complex required for kinetochore functions.. Mol Biol Cell.

[30] Starr DA, Saffery R, Li Z, Simpson AE, Choo KH (2000). HZwint-1, a novel human kinetochore component that interacts with HZW10.. J Cell Sci.

[31] Starr DA, Williams BC, Hays TS, Goldberg ML (1998). ZW10 helps recruit dynactin and dynein to the kinetochore.. J Cell Biol.

[32] Yang ZY, Guo J, Li N, Qian M, Wang SN (2003). Mitosin/CENP-F is a conserved kinetochore protein subjected to cytoplasmic dynein-mediated poleward transport.. Cell Res.

[33] Kops GJ, Kim Y, Weaver BA, Mao Y, McLeod I (2005). ZW10 links mitotic checkpoint signaling to the structural kinetochore.. J Cell Biol.

[34] Yan X, Li F, Liang Y, Shen Y, Zhao X (2003). Human Nudel and NudE as regulators of cytoplasmic dynein in poleward protein transport along the mitotic spindle.. Mol Cell Biol.

[35] Fujiwara T, Takami N, Misumi Y, Ikehara Y (1998). Nordihydroguaiaretic acid blocks protein transport in the secretory pathway causing redistribution of Golgi proteins into the endoplasmic reticulum.. J Biol Chem.

[36] Nakamura M, Nakazawa J, Usui T, Osada H, Kono Y (2003). Nordihydroguaiaretic acid, of a new family of microtubule-stabilizing agents, shows effects differentiated from paclitaxel.. Biosci Biotechnol Biochem.

[37] Shah JV, Botvinick E, Bonday Z, Furnari F, Berns M (2004). Dynamics of centromere and kinetochore proteins; implications for checkpoint signaling and silencing.. Curr Biol.

[38] Howell BJ, Moree B, Farrar EM, Stewart S, Fang G (2004). Spindle checkpoint protein dynamics at kinetochores in living cells.. Curr Biol.

[39] Kallio MJ, Beardmore VA, Weinstein J, Gorbsky GJ (2002). Rapid microtubule-independent dynamics of Cdc20 at kinetochores and centrosomes in mammalian cells.. J Cell Biol.

[40] Buffin E, Lefebvre C, Huang J, Gagou ME, Karess RE (2005). Recruitment of Mad2 to the kinetochore requires the Rod/Zw10 complex.. Curr Biol.

[41] Waters JC, Chen RH, Murray AW, Salmon ED (1998). Localization of Mad2 to kinetochores depends on microtubule attachment, not tension.. J Cell Biol.

[42] Li D, Morley G, Whitaker M, Huang JY (2010). Recruitment of Cdc20 to the kinetochore requires BubR1 but not Mad2 in Drosophila melanogaster.. Mol Cell Biol.

[43] Rahmani Z, Gagou ME, Lefebvre C, Emre D, Karess RE (2009). Separating the spindle, checkpoint, and timer functions of BubR1.. J Cell Biol.

[44] Hori T, Haraguchi T, Hiraoka Y, Kimura H, Fukagawa T (2003). Dynamic behavior of Nuf2-Hec1 complex that localizes to the centrosome and centromere and is essential for mitotic progression in vertebrate cells.. J Cell Sci.

[45] Kishi K, van Vugt MA, Okamoto K, Hayashi Y, Yaffe MB (2009). Functional dynamics of Polo-like kinase 1 at the centrosome.. Mol Cell Biol.

[46] Delacour-Larose M, Molla A, Skoufias DA, Margolis RL, Dimitrov S (2004). Distinct dynamics of Aurora B and Survivin during mitosis.. Cell Cycle.

[47] Delacour-Larose M, Thi MN, Dimitrov S, Molla A (2007). Role of survivin phosphorylation by aurora B in mitosis.. Cell Cycle.

[48] Chan GK, Schaar BT, Yen TJ (1998). Characterization of the kinetochore binding domain of CENP-E reveals interactions with the kinetochore proteins CENP-F and hBUBR1.. J Cell Biol.

[49] Chan GK, Jablonski SA, Sudakin V, Hittle JC, Yen TJ (1999). Human BUBR1 is a mitotic checkpoint kinase that monitors CENP-E functions at kinetochores and binds the cyclosome/APC.. J Cell Biol.

[50] Jablonski SA, Chan GK, Cooke CA, Earnshaw WC, Yen TJ (1998). The hBUB1 and hBUBR1 kinases sequentially assemble onto kinetochores during prophase with hBUBR1 concentrating at the kinetochore plates in mitosis.. Chromosoma.

[51] Liu ST, Chan GK, Hittle JC, Fujii G, Lees E (2003). Human MPS1 kinase is required for mitotic arrest induced by the loss of CENP-E from kinetochores.. Mol Biol Cell.

[52] Campbell MS, Chan GK, Yen TJ (2001). Mitotic checkpoint proteins HsMAD1 and HsMAD2 are associated with nuclear pore complexes in interphase.. J Cell Sci.

[53] Sudakin V, Chan GK, Yen TJ (2001). Checkpoint inhibition of the APC/C in HeLa cells is mediated by a complex of BUBR1, BUB3, CDC20, and MAD2.. J Cell Biol.

