# Correction: Dynein/Dynactin-Mediated Transport of Kinetochore Components off Kinetochores and onto Spindle Poles Induced by Nordihydroguaiaretic Acid

**DOI:** 10.1371/annotation/272736a3-6a7a-44ba-bbae-fd5d7e97d651

**Published:** 2011-02-17

**Authors:** Jakub K. Famulski, Larissa J. Vos, Jerome B. Rattner, Gordon K. Chan

The current address symbol was not included after the first author's name. The current address, "Department of Biological Sciences, University of Alberta, Alberta, Canada," applies to Jakub K. Famulski.

