# Supplementary figures and images for: Dynein/Dynactin-Mediated Transport of Kinetochore Components off Kinetochores and onto Spindle Poles Induced by Nordihydroguaiaretic Acid
Source: PLoS One. 2011 Jan 28;6(1):e16494. doi: 10.1371/journal.pone.0016494 (PMC3030593; doi:10.1371/journal.pone.0016494)

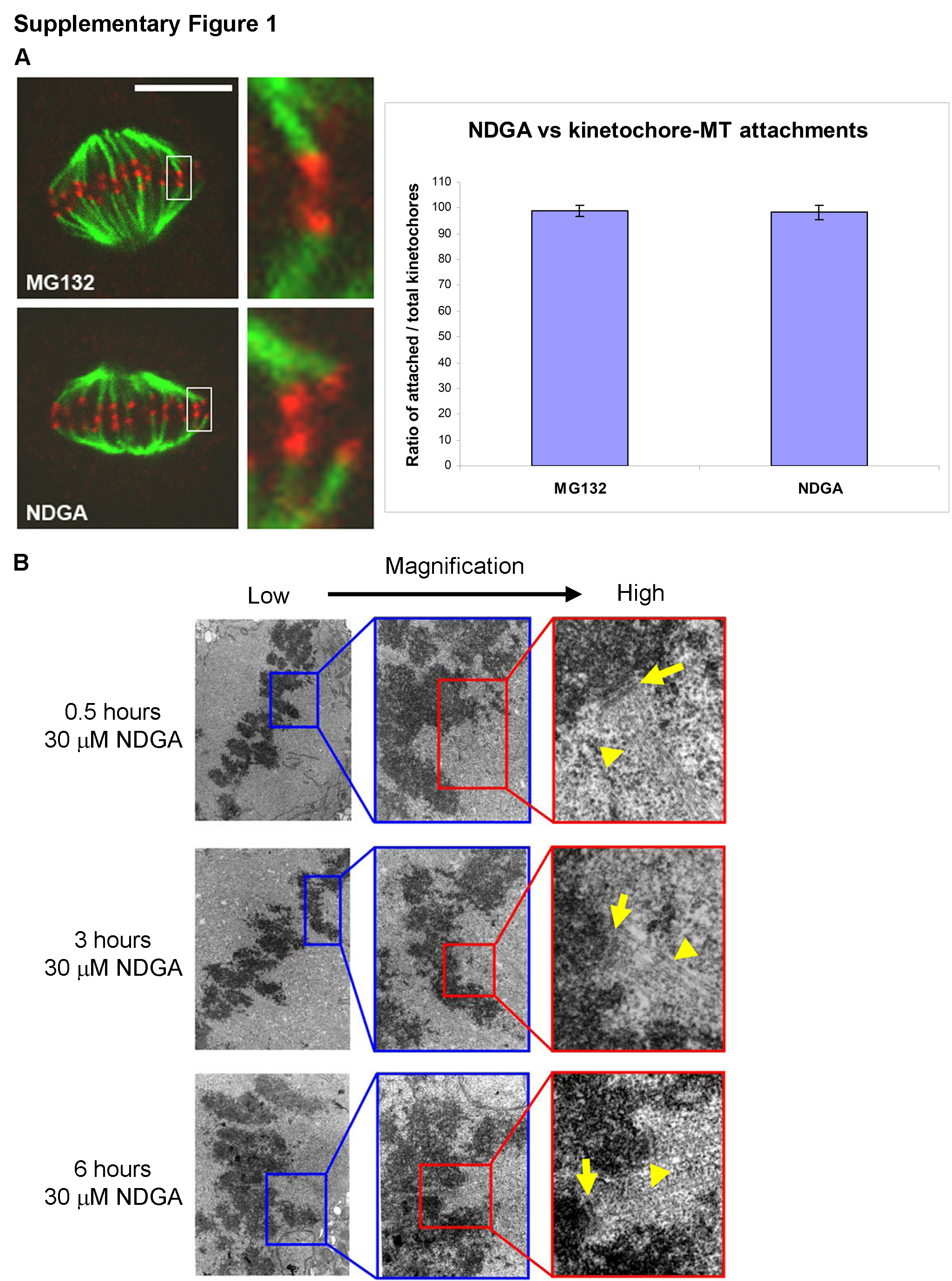

Supplement: Figure S1 — NDGA treatment does not disrupt k-MT attachments. A) HeLa cells arrested with 12.5 µM MG132 and subsequently treated with NDGA were exposed to ice cold media for 10 minutes and harvested, immunofluorescence stained and imaged in 3D using confocal microscopy. Tubulin and ACA staining shows that NDGA does not affect k-MT attachments. Insets show enlargements of k-MT attachments. Tubulin is shown in green, ACA in red. Scale bar = 10 µm. ∼50 kinetochores per cell, as observed by ACA staining, were analyzed for MT attachments and scored as ratio of attached/total. MG132 n = 3 cells, 164 kinetochores and NDGA n = 10 cells, 490 kinetochores. Error bars = +/− one standard deviation. B) HeLa cells treated with NDGA for 0.5 hours (top), 3 hours (middle) or 6 hours (bottom) were fixed and analyzed for k-MT attachments using electron microscopy. Shown are three different magnifications of chromosomes and their corresponding k-MTs. In all three NDGA treatments normal kinetochore plates as well as k-MTs are observed. Yellow arrows indicate kinetochores while the arrow heads indicate k-MTs. (TIF) [file pone.0016494.s001.tif]

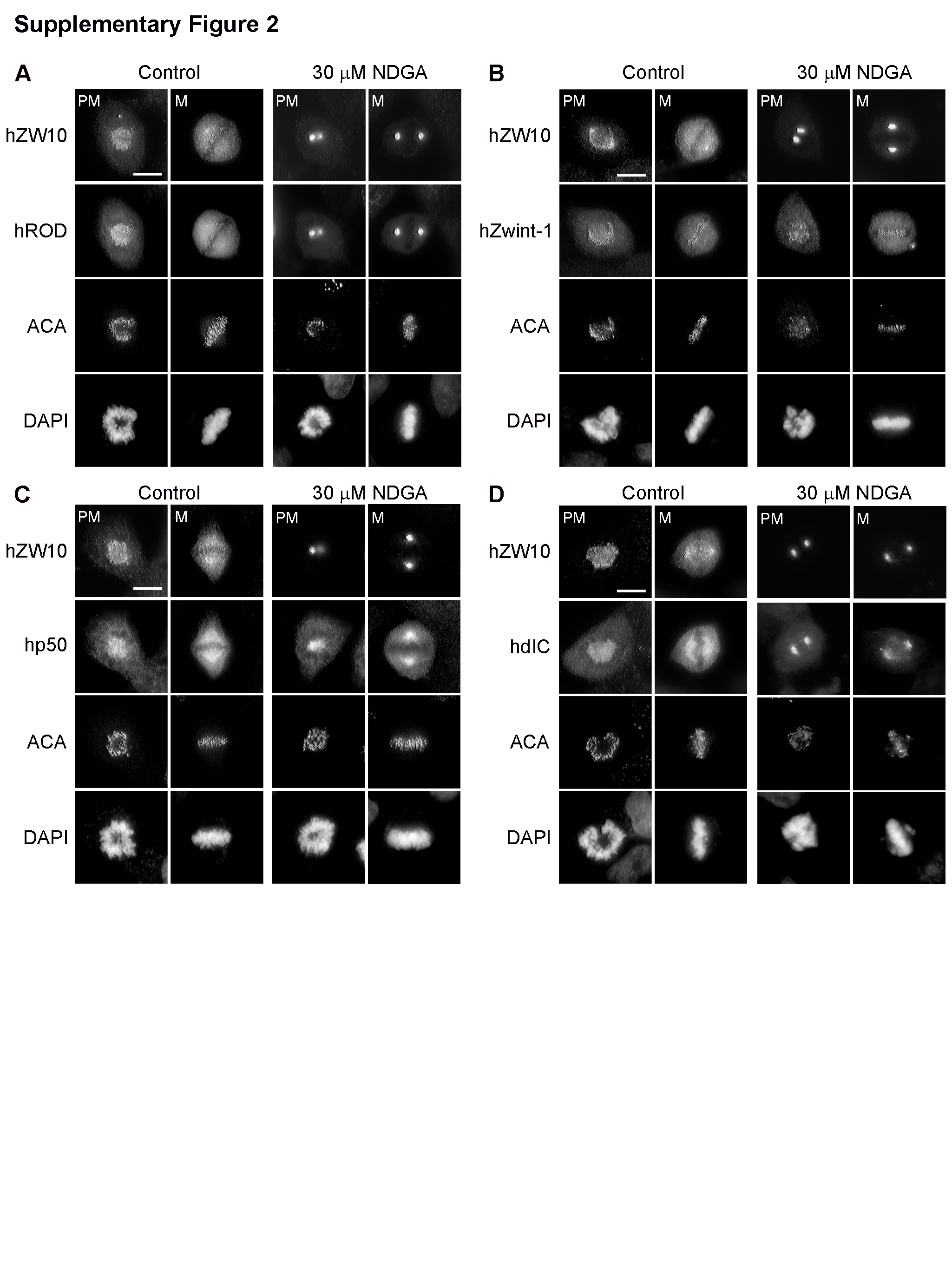

Supplement: Figure S2 — NDGA transport of hZW10, hROD, hdIC and hp50. A-D) HeLa cells treated with 30 µM NDGA for 30 minutes and stained with hZW10, ACA and either: hROD (A), hZwint-1 (B), hp50 (C) or hdIC (D) antibodies. hZW10, hROD, hdIC and hp50 are observed to accumulate at spindle poles while hZwint-1 does not. Chromosomes are stained with DAPI. Scale bar = 10 µm. (TIF) [file pone.0016494.s002.tif]

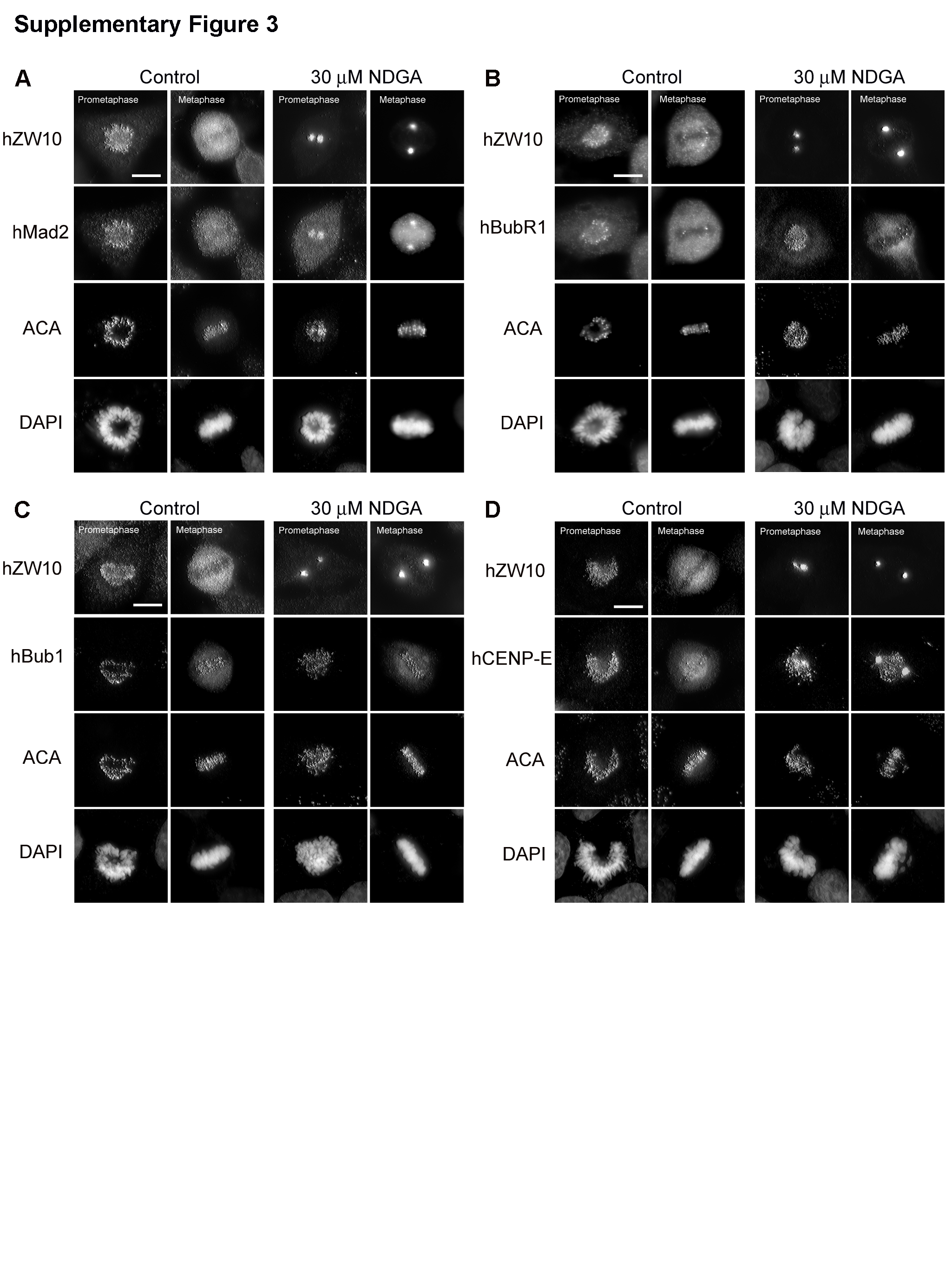

Supplement: Figure S3 — hMad2 and hCENP-E but not hBub1 or hBubR1 are transported to spindle poles in the presence of NDGA. A–D) HeLa cells treated with 30 µM NDGA for 30 minutes and stained with hZW10, ACA and either: hMad2 (A), hBubR1 (B), hBub1 (C) or hCENP-E (D) antibodies. hZW10, hCENP-E and hMad2 are observed to accumulate at spindle poles while hBub1 and hBubR1 do not. Chromosomes are stained with DAPI. Scale bar = 10 µm. (TIF) [file pone.0016494.s003.tif]

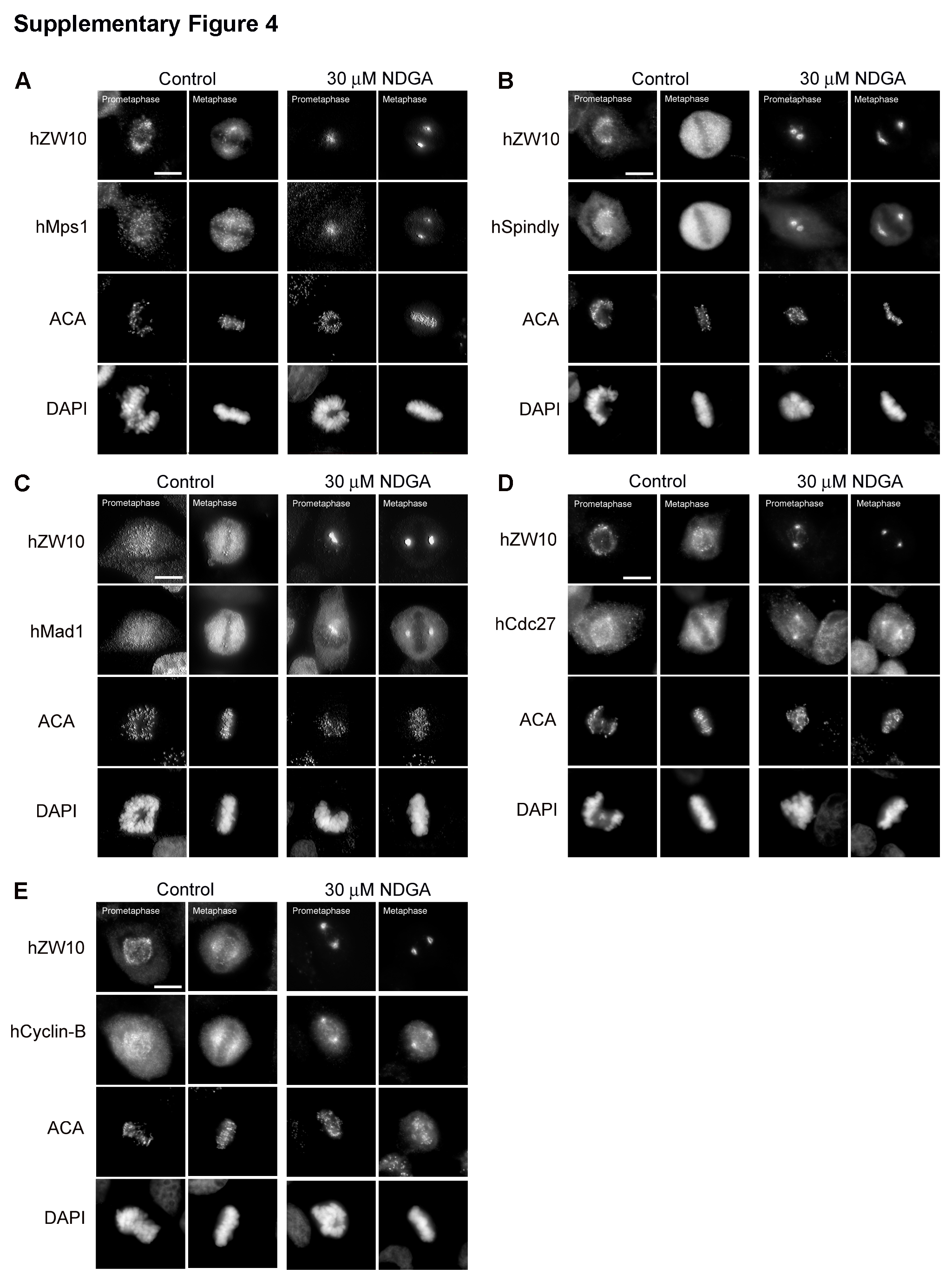

Supplement: Figure S4 — hMps1, hSpindly, hMad1, Cdc27 and cyclin- B are transported to spindle poles in the presence of NDGA. A–E) HeLa cells treated with 30 µM NDGA for 30 minutes and stained with hZW10, ACA and either: hMps1 (A), hSpindly (B), hMad1 (C), hCdc27 (D) or cyclin-B (E) antibodies. hZW10, hMps1, hMad1, hCdc27, cyclin-B and hSpindly are observed to accumulate at spindle poles. Chromosomes are stained with DAPI. Scale bar = 10 µm. (TIF) [file pone.0016494.s004.tif]

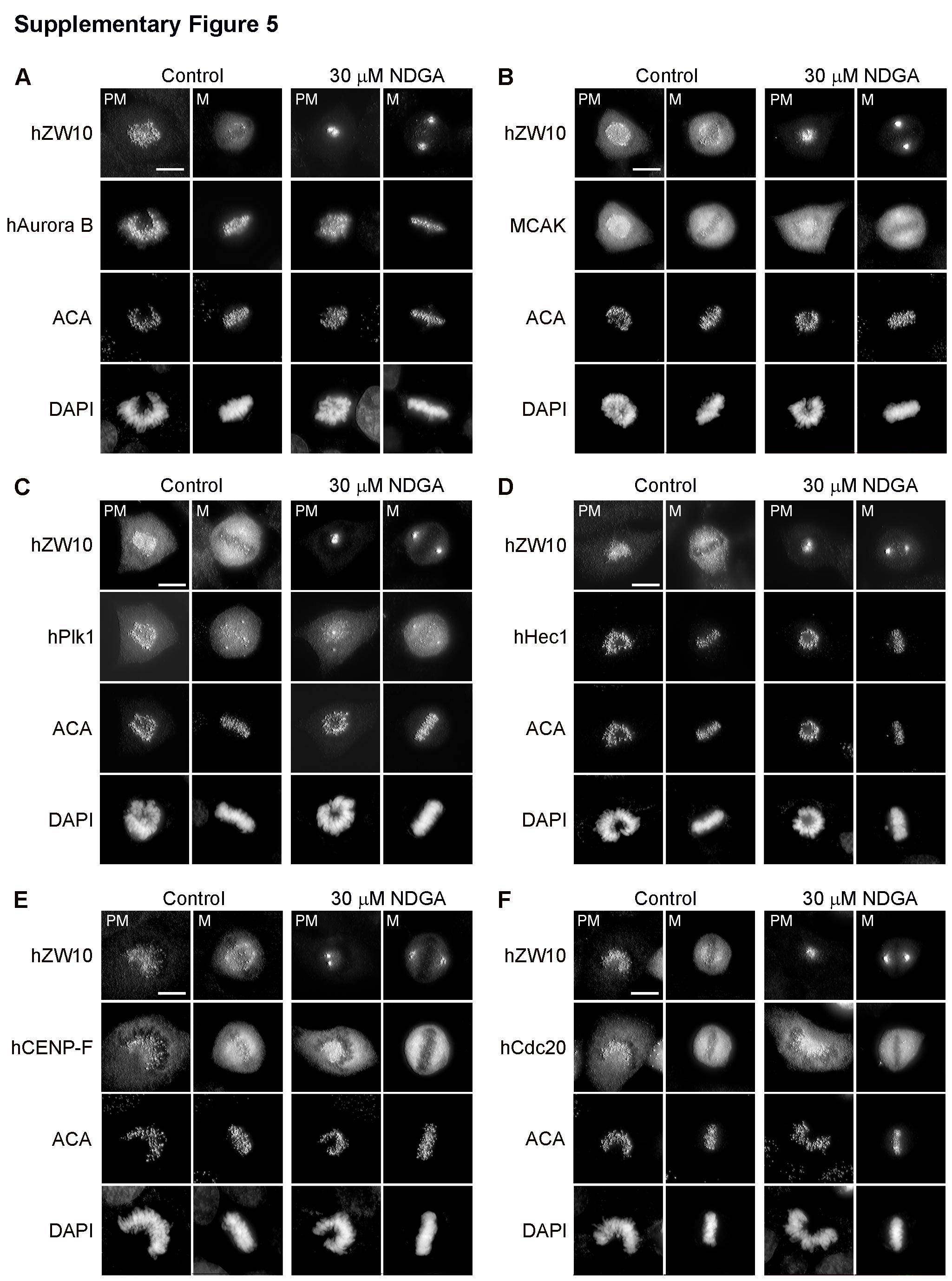

Supplement: Figure S5 — hAurora B, MCAK, hPlk1, hHec1, hCENP-F and hCdc20 are not transported to spindle poles in the presence of NDGA. A–F) HeLa cells treated with 30 µM NDGA for 30 minutes and stained with hZW10, ACA and either: hAurora B (A), MCAK (B), hPlk1 (C), hHec1 (D), hCENP-F (E) or hCdc20 (F) antibodies. Only hZW10 is observed to accumulate at spindle poles. Chromosomes are stained with DAPI. Scale bar = 10 µm. (TIF) [file pone.0016494.s005.tif]

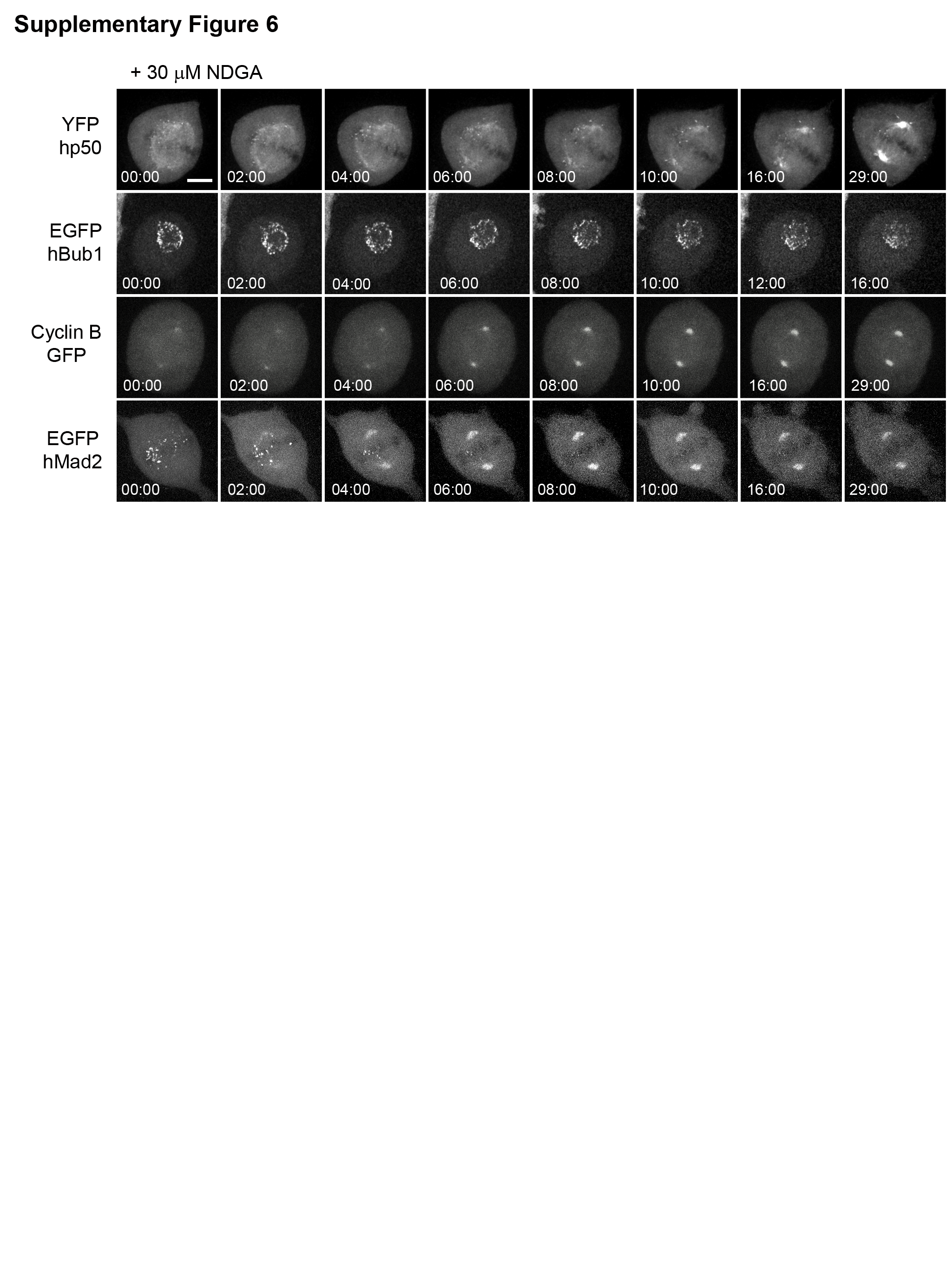

Supplement: Figure S6 — Live cell analysis of hp50, hBub1, cyclin- B and hMad2 response to NDGA treatment. HeLa cells transiently transfected with either: YFP-hp50, EGFP-hBub1, cyclin-B-GFP or EGFP-hMad2 were imaged using a spinning disk confocal microscope. Upon addition of NDGA, the cells were imaged every 1 minute as a Z-stack of ∼20 images 1 µm apart. Maximum projections are shown. YFP-hp50, cyclin-B-GFP and EGFP-hMad2 are observed to accumulate at spindle poles upon NDGA treatment while EGFP-hBub1 does not. Time is indicated as minutes:seconds. Scale bar = 10 µm. (TIF) [file pone.0016494.s006.tif]
